# Skin and gill microbiome profiles and network structures in farmed tilapia (*Oreochromis niloticus*) and their relationships with health conditions

**DOI:** 10.1186/s42523-025-00480-2

**Published:** 2025-10-29

**Authors:** Sanjit C. Debnath, Ashley G. Bell, Jamie McMurtrie, Ben Temperton, Charles R. Tyler

**Affiliations:** 1 Faculty of Health and Life Sciences, University of Exeter, Exeter, Devon, EX4 4QD UK; 2 Sustainable Aquaculture Futures, University of Exeter, Exeter, Devon, EX4 4QD UK; 3 Section of Nutrition, Department of Metabolism, Digestion and Reproduction, Faculty of Medicine, Imperial College London, Hammersmith Campus, Du Cane Road, London, W12 0NN UK

**Keywords:** Tilapia (*Oreochromis niloticus*), Skin microbiome, Gill microbiome, Fish disease, Co-occurrence network, Geographic locations

## Abstract

**Background:**

Tilapia is one of the most popular finfish in aquaculture, but various emerging infectious diseases are limiting the growth of the tilapia aquaculture industry globally. The external mucosal microbiomes of fish act as a first line of defence for maintaining host health. However, how skin and gill microbiomes differ between healthy and naturally infected tilapia remains poorly understood. Here, we employed 16S rRNA and 18S rRNA high-throughput metabarcoding to characterise the microbiome of tilapia skin, gills, and water from ponds reported with diseased and non-diseased conditions, and to investigate signatures of microbial dysbiosis related to health conditions.

**Results:**

Microbial diversity varied significantly across different sample types (gill, skin and pond water) and geographical locations. Skin and gill microbiomes from reported non-diseased conditions differed in the presence of the commensal genus *Cetobacterium*, while diseased gill-skin were enriched with pathogenic genera including *Flavobacterium*, *Aeromonas*,* Vibrio*, *Vogesella*, and *Klebsiella*. Additionally, the relative abundance of diatom *Cyclotella* in pond water under diseased conditions appeared to be almost double that of non-diseased pond water, albeit this was statistically non-significant. *Cetobacterium* formed a core component of the bacterial genera in the non-diseased gill and skin microbiome. In contrast, *Aeromonas* formed a core component of the core microbiome in the diseased gill and skin microbiomes. Analysis of the microbial co-occurrence network in the diseased skin and gill found it to be relatively less complex compared with these tissues in the non-diseased state.

**Conclusions:**

The findings show that the tilapia microbiome differs across the skin and gill tissue surfaces, and from the pond waters in which they are cultured. In reported diseased cases, these microbiomes show enrichment of potential pathogenic genera and less complex microbial co-occurrence networks, which may be used as an indicator of microbial dysbiosis in aquaculture systems. Understanding how these alterations may be used to predict potential disease outbreaks requires an understanding of the functional impacts of the changes in the microbial assemblages, allowing for timely interventions to mitigate the impacts of disease in the aquaculture system.

**Graphical Abstract:**

**Figure:**
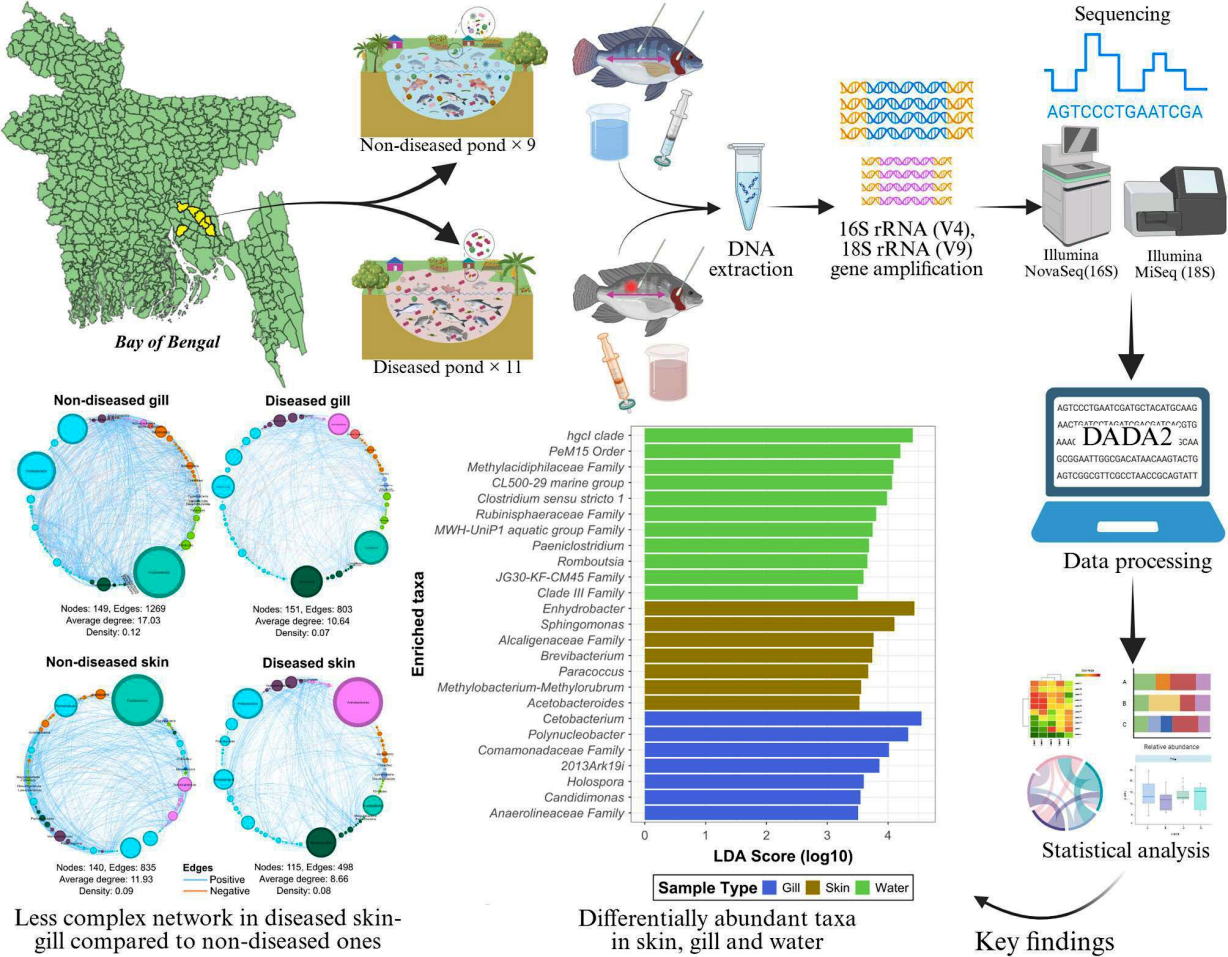

**Supplementary Information:**

The online version contains supplementary material available at 10.1186/s42523-025-00480-2.

## Background

Aquaculture is one of the fastest-growing food production sectors that contributes substantially to global food security, supplying millions of tonnes of aquatic products annually [1, 2]. To help meet the global demand for animal protein, aquaculture is shifting from extensive to semi-intensive and intensive culture systems. Intensification, however, tends to result in reduced water quality, which can stress the cultured organisms and increase susceptibility to diseases [3–7]. Understanding relationships between the microbiome(s) in aquaculture environments and the host species could help in understanding disease susceptibility and resilience, as well as identifying markers of tipping points for disease onset for use in disease management and mitigation strategies in the aquaculture system [8]. Various studies have been conducted to help understand how various factors including disease affect fish microbiomes [9–11]. However, the majority of these have been on the gut microbiome with much less known for the skin and gill mucosal layers of fish [12]. Furthermore, the external skin and gill surfaces of fish are in intimate contact with the surrounding water, and there is a continuous exchange of microbial communities between these surfaces and the water environment that includes pathogens [13]. Protection against invading pathogens at these epithelial surfaces includes via mucus produced from goblet cells that contain various immunogenic compounds including antimicrobial peptides, proteases, lysozymes and lectins [12, 14–16]. Commensal microorganisms in the mucosal surfaces also limit the colonisation of pathogenic microorganisms by producing friction-preventing polymers and complex inhibitory antimicrobials [17–20], and also entrapping the pathogens and sloughing them from the skin/gill surfaces [12, 15, 21, 22].

Any disruption in the normally balanced interactions between the host, the environment, and associated microorganisms can lead to the invasion of pathogenic bacteria and subsequent disease outbreaks [23]. Various stressors such as hypoxia and high stocking density [24], microparasitic (bacterial, viral and parasitic) infections [25–27] and/or water conditions [28] are known to disturb microbial homeostasis, known as dysbiosis. During dysbiosis, a shift in microbial composition, diversity and functionality can occur [29], resulting in the proliferation of opportunistic pathogens, which in turn influence the host physiology (including the immune system), potentially leading to disease outbreak(s) [23]. A common finding in a diseased state is a decrease in the abundance and diversity of commensal/beneficial microbes. Microbial dysbiosis caused either by environmental stressors and/or bacterial/viral infections, can also make fish more susceptible to secondary bacterial (or other disease-causing organism) infection [27, 30]. The dynamics of the gill and skin microbiomes of fish, in turn, may provide a useful indicator of the health status of the fish. Understanding the microbiomes that act to buffer against disease-causing pathogens (existing and emerging) thus has the potential to help reduce infectious diseases in aquaculture.

Tilapia are one of the most widely farmed finfish species globally. Asian countries produce most of the world’s tilapia [31] and are also the major consumers of this fish species [32]. In Bangladesh, tilapia is the third most farmed fish and is often grown in polyculture systems (culturing various species together). The species also has good adaptability to poor water quality, is a low-tropic species, and has relatively high resistance to stress and disease. However, due to intensification and other stressors, tilapia are succumbing to various existing and emerging diseases [33], causing millions of dollars in losses each year. Recently, various studies have investigated the skin and gill microbiomes of several fish species focused on microbial dysbiosis resulting from induced infection by pathogenic bacteria [12, 15, 26, 34–36]. There are very few studies, however, that have focused on comparing the microbial communities in healthy and infected tilapia [37, 38]. To better understand the role of microbiomes in maintaining tilapia health and assessing for microbial signatures of dysbiosis that may signal disease onset, it is necessary to understand the microbial community structure and diversity in these fish in non-diseased and diseased states in the systems in which they are cultured.

In this study, we applied 16S and 18S rRNA high-throughput amplicon sequencing to characterise the microbial communities from the gill and skin of tilapia and the surrounding water from 11 reported diseased and 9 non-diseased semi-intensive polyculture ponds located in seven upazilas of the Cumilla and Chandpur districts in Bangladesh (see Additional file 1, Fig. S1). Cumilla and Chandpur are some of the most important districts for inland fish production, producing nearly 0.31 million tonnes and 0.11 million tonnes finfish, respectively [39]. This study aimed to assess: (i) if the microbial composition and diversity differ across tilapia skin, gill and pond water and if within each sample type, there is a difference between diseased and non-diseased earthen pond systems; (ii) how reported disease state influence microbial co-occurrence network; (iii) assess the dysbiosis patterns in bacterial genera that may signal for diseased states, and; (iv) if so, how this differed across different geographical regions.

## Methods

### Sample collection for microbiome analysis

Tilapia gill swabs (*n* = 121), skin swabs (*n* = 121) from 121 tilapia and pond water (*n* = 60) samples from 20 earthen ponds practising semi-intensive polyculture of tilapia with various mixed finfish species were collected between November 2020 and October 2021. Nine of these ponds had no obvious signs of infection or a health problem for at least three months before the sampling and were designated as the non-diseased group. Samples were collected from eleven ponds that contained fish that had some form of disease as determined by major external skin lesions and /or included dying fish, designated as the diseased group. The disease status was based on farmer reports and then field identification of external signs of a diseased state for individual fish via the presence of external lesions/infections and/or moribund fish by the lead researcher, but without any molecular or histopathological confirmation for any specific pathogens. We classified the “diseased” fish with caution, referring to them as “reported diseased fish” in the absence of any definitive diagnoses the causative pathogens or specific disease. These ponds were located at seven different upazilas in the Cumilla and Chandpur districts in Bangladesh (Additional file 1, Fig. S1). Geographical distance from the first sampling pond (P1) to others ranged from 19 km to around 60 km. Between six and eight fish (except for pond P9) were captured, from which skin and gill swabs were collected. Surface water was also collected from three different locations for each pond (Additional file 2, Table S1). The ponds range in size between 728 m^2^ and 263,046 m^2^ and the fish were between 3 and 4 months old in age (Additional file 2, Table S1). Groundwater and rainwater were the main water sources. Floating commercial feed was used unless otherwise stated (Additional file 2, Table S1).

To collect the microbial biomass from the fish’s skin and gill, these surfaces were swabbed three times with sterile polyester swabs (Deltalab, Spain) following the methods of Delamare-Deboutteville et al. [40]. To obtain pond water microbial samples, the surface water was collected from three different locations (of each pond) and passed through a polycarbonate membrane 0.4 μm filter (MerckMillipore, United States) using a 200 ml syringe. Filtration was performed until the filter was blocked and no more water could be pushed through the filter; this resulted in a variable volume being filtered of between 48 and 180 mL (depending on the number of suspended particles in the pond water; see Additional file 2, Table S1). Swabs and the filter were transferred immediately in molecular-grade ethanol in 2 mL cryogenic screw-cap tubes. A total of 302 samples were collected for prokaryotic community analysis and 60 samples for microeukaryotic community analysis, across all ponds (Additional file 2, Table S1). All the samples were stored at room temperature until they could be placed into a − 20 °C freezer in the WorldFish lab in Bangladesh. On arrival in the UK, they were stored at − 80 °C until processing for DNA extraction.

### DNA extraction, library preparation and sequencing

For DNA extraction, the ethanol was first removed using a geneVac EZ-2 Evaporator and thereafter, the samples were stored at − 80 °C. DNA extraction was performed through a CTAB/EDTA/chloroform method as previously described [30]. The detailed protocol is available at 10.17504/protocols.io.bw8gphtw. After extraction, using the Promega QuantiFluor ONE dsDNA quantification kit (E4870) DNA concentration was quantified with the Promega GloMax instrument.

To amplify the small subunit ribosomal ribonucleic acid (SSU rRNA) marker genes of the prokaryotes and microeukaryotes from the collected samples, polymerase chain reaction (PCR) was performed using the Earth Microbiome Project recommended primers. A one-step PCR was performed to amplify the 18S rRNA V9 hypervariable region using 1391f 5′-GTACACACCGCCCGTC-3′ [41] and EukBr 5′-TGATCCTTCTGCAGGTTCACCTAC-3′ [42] with a dual-indexing scheme [43]. For the amplification of 16S rRNA V4 region, primer pairs 515F (Parada) 5’-GTGYCAGCMGCCGCGGTAA-3′ [44] and 806R (Apprill) 5’-GGACTACNVGGGTWTCTAAT-3′ [45] were synthesised using updated 16S primers, barcodes, and linkers from the Earth Microbiome Project [46–48], and each well of each plate was dual-indexed to minimise index hopping during sequencing. After the PCR amplification of V4 regions, each plate was pooled in equimolar amounts, and an Illumina barcode was ligated to the pooled library, enabling the use of multiple plates with identical indexes, as has been previously performed [49].

For gill and skin swab samples, the PCR thermocycling conditions for amplifying the 16S V4 region were as follows: initial denaturation at 98 °C for 30s, followed by 30 cycles of denaturation at 98 °C for 10s, annealing step at 61 °C for 20s, extension at 72 °C for 30s and then a final extension of 72 °C for 2 min. For the water samples, the number of cycles was reduced to 27 (versus 30) cycles to minimise the chance of the amplification of non-target material. Individual samples within each PCR library were run as 50 µL reactions with 25 µL Platinum SuperFi II PCR Master Mix (Thermo Fisher Scientific), 2.5 µL of 10 µM forward and reverse primers (final concentration 0.5 µM), and 20 µL of 25ng/µL DNA (final concentration 500 ng) for swabs and 4 µL of 2.5ng/µL DNA (final concentration 10 ng) for water samples. For the 18S V9 amplification, the conditions were as follows: initial denaturation at 98 °C for 30s, followed by 28 cycles of denaturation at 98 °C for 10s, annealing step at 63 °C for 20s, extension at 72 °C for 30s and then a final extension of 72 °C for 2 min. For the V9 region of the 18S rRNA, 25 µL NEBNext High-Fidelity PCR Master Mix (New England Biolabs), was used rather than Platinum SuperFi II PCR Master Mix and a total of 15 ng DNA template was used for each sample. For each 96-plate library, minimally two extraction controls, one PCR blank and one mock community DNA standard and/or extract (ZymoBIOMICS^®^ Microbial Community DNA standard) were included. The amplification was visualised with 1% agarose gel electrophoresis. PCR amplicons were purified by removing primer dimers from the amplicons using Agencourt AMPureXP magnetic beads (Beckman Coulter, USA). The ratio of the AMPureXP magnetic beads reagent to each amplicon was set to 0.8 (v/v) in the purification process and this was washed twice with 200 µL of freshly made 80% ethanol for 2 min. After the final wash, beads were dried at room temperature for 5 min, then eluted with 18 µL elution buffer (EB, 10 mM Tris-Cl, pH 8.5 Sigma) mixed with 10% (weight/volume) Tween. After purification, amplicon libraries were quantified using the Promega QuantiFluor ONE dsDNA quantification system (Promega, USA) and pooled at an equimolar ratio. Subsequently, the samples were sequenced by the University of Exeter Sequencing Service on the Illumina NovaSeq platform, using 250 bp paired-end for prokaryotic (16S) and on the Illumina Miseq platform using v2 chemistry and 150 bp paired-end for microeukaryotic (18S).

### Bioinformatics and data analysis

#### Raw data processing and taxonomic assignment

After sequencing, the 18S rRNA amplicon sequences were demultiplexed by the Exeter sequencing centre. The 16S rRNA amplicon sequences were demultiplexed using Illumina barcodes and using the Cutadapt algorithm v4.5 [50]. They were demultiplexed to the sample level, keeping the reads and the dual indexes. Nucleotides with a quality score of less than or equal to Q2, containing “N”s as base pairs were removed. After demultiplexing, the read quality for each sample was checked through the DADA2 pipeline v1.26.0 [51]. For prokaryotes, based on an overall quality threshold of 30, the forward and reverse reads were truncated at positions 216 and reverse reads at 217. For the microeukaryotes, the forward and reverse reads were truncated, with the overall quality score being >Q30 throughout. Amplicon sequence variants (ASVs) were subsequently inferred. To obtain the full denoised sequences, the forward and reverse reads were merged, providing both reads overlapped with a minimum of 12 bp for both prokaryotes and microeukaryotes. Only ASVs between 252 and 254 bp in size for prokaryotes, and between 103 and 133 bp for microeukaryotes, were retained to avoid any off-target sequencing effect, and any chimaeras were removed. After this step, sequences with removed off-target and chimaeras were merged, used to construct the ASV table, and using the *assignTaxonomy* function from DADA2, taxonomy for the prokaryotes dataset was assigned to all ASVs against the SILVA SSU V138.1 taxonomic database [52]. For the microeukaryotes, taxonomy was assigned to each ASV using the PR2 v5.0.0 taxonomic database [53]. To ensure even DNA extraction and accuracy of the taxonomic assignment, microbial communities in sequenced positive controls (ZymoBIOMICS^®^ Microbial Community DNA standard) were compared against the manufacturer’s expected microbial community composition (Additional file 1, Fig. S2). All bioinformatics as well as statistical analysis were performed in RStudio v2023.06.0 + 421 using R (version 4.3.3).

#### Phylogenetic tree construction

To construct a phylogenetic tree of ASVs, sequences were aligned with MAFFT v7.475 [54] before determining the best-fitting tree model with ModelTest-NG v0.1.7 [55]. Model selection was made according to the lowest-scoring Akaike information criterion (AIC) and Bayesian information criterion (BIC) scores. The final phylogenetic tree was constructed with IQTREE v2.1.2 [56] using a GTR + I + G4 model, and the tree was rooted using the longest terminal branch as an outgroup.

#### Phyloseq object construction

An ASV table representing amplicon sequence variants, a taxonomy table providing taxonomic assignments produced by the DADA2 pipeline, a phylogenetic tree elucidating evolutionary relationships among microbes and a sample metadata table were amalgamated to construct a phyloseq object. This phyloseq object was later used for further quality control and statistical analysis. Low abundance ASVs were removed, and ASVs present in at least 2 (prevalence threshold >= 2) samples and with a total abundance greater than or equal to 30 per sample were retained. Likely contaminants were identified using the decontam package v1.18.0 [57] with a prevalence threshold = 0.5 and these were removed before further analysis. For the 16S rRNA data, any ASVs assigned as Chloroplast (rank = Order), Mitochondria (rank = Family), Eukaryota, Archaea, or unclassified (NA) at the Kingdom level were discarded. For microeukaryotes, any ASVs identified as Craniata (rank = Class), Teleostei (rank = Family), bacteria or unclassified (NA) at the domain level were removed to increase the accuracy and interpretability of subsequent analyses [51, 58]. Samples with less than 2000 reads were also removed before further analysis. After all the filtering, for 16S there were 298 remaining samples out of 302 and for 18S there were 57 remaining samples out of 60 for the downstream analysis.

### Statistical analysis

#### Microbial diversity

Microbial alpha diversity (within samples) was estimated at the ASV level using Chao1 (that estimates the species richness i.e., number of species, including rare species) [59], Shannon (that measures both the richness and evenness of the taxa present and considers the abundance of each species) [60], and Faith’s phylogenetic (PD) diversity (that takes into account the species phylogenetic relationship and uses the evolutionary distance to calculate a sample’s diversity) [61] indices applying phyloseq and picante packages in R [62, 63]. To assess the univariate influence of sample types, reported disease state and geographical locations on the alpha diversity indices (Chao1, Shannon and PD), we initially used Kruskal–Wallis (KW) tests followed by pairwise testing using the post-hoc Dunn’s test with Benjamini-Hochberg (BH) adjustment for controlling false discovery rate (FDR) for multiple testing. To account for the repeated sampling from ponds across geographical locations, based on the residual distributions, we applied a Linear Mixed Effects model (LMM) with LmerTest package v3.1.1 [64] for Chao1 and PD, while Shannon was investigated using a Generalised Mixed Effects model (GLMM) with Gamma distribution with GlmmTMB package v1.1.11 [65] in R. Since we wanted to investigate whether microbial alpha diversity varies across different sample types (predictor) and within each sample type, whether reported disease (predictor) influence alpha diversity, and whether geographical locations also contribute to the difference in alpha diversity, we used sample type, reported disease and their interaction (sample type × reported disease) and location (Upazila) as fixed effects and pond (20 aquaculture pond) as a random factor, to account for the repeated sampling within the ponds. Therefore, the final model (LMM/GLMM) was: alpha diversity ~ Sample type × Reported disease + Upazila + (1 | Pond). The normality of the residuals was checked using the Q-Q plot and heteroskedasticity (fitted vs. residuals) plots. To check the significance of the fixed effects and their interaction, a Type III chi-square ANOVA test was performed, and pairwise comparisons of the estimated marginal means were assessed using the emmeans R package v1.11.1 [66] with BH adjustment as mentioned above.

Beta diversity for investigating microbial structures in different sample groups (sample type, reported disease, geographical locations) was calculated following the Bray-Curtis distance using phyloseq package [62], vegan v2.6.4 [67] and visualised using microViz v0.10.10 [68] packages in R. Pairwise dissimilarities between different samples were calculated through Principal coordinate analysis (PCoA), using a Bray-Curtis dissimilarity metric at the ASVs level and visualised using ggplot2 [69] and microViz [68] packages. A PERMANOVA (permutational multivariate statistical analysis of community separation) test [70] was carried out using the *adonis2* function of the vegan R package [67], and if there was a significant difference (*p* < 0.05), a pairwise comparison using *pairwise.adonis* function from the pairwiseAdonis R package v0.4.1 was used [71] to check if the variance of taxonomic compositions differed between different groups. To assess the combined effects of reported disease and geographical locations on the overall microbial community structure, we used a PERMANOVA model using adonis2(bray distance ~ Reported disease + Upazila, data = metadata, permutations = 999, strata = metadata$Pond name).

#### Core microbiome

To investigate what might constitute the core microbiota of the tilapia gill and skin, core microbiomes were assessed for each surface on the ASV-level compositional data using the *microbiome* R package v1.24.0 [72]. For each tissue sample type (gill and skin swabs), core taxa were identified with a detection threshold of 0.01% relative abundance and a minimum prevalence of 90%. Therefore, to be considered part of the core microbiome, taxa must have a relative abundance greater than 0.01% in at least one sample and be present in at least 90% of all samples within each sample type. Identified core ASVs for gill and skin swab samples were aggregated to the genus level and visualised using the microViz package.

#### Differential abundance analysis

To identify differences in bacteria of biological relevance between different fish tissues (gill and skin) and pond water for diseased versus non-diseased ponds, linear discriminant analysis (LDA) effect size (LEfSe) was performed at the genus level [73] using microbiomeMarker v1.8.0 R package [74]. Differential taxa were defined as those having a *p*-value less than 0.05 determined by both the Kruskal–Wallis rank sum test, followed by pairwise Wilcoxon rank-sum test, and those with an LDA cut-off value (log-transformed) of 3.5, without adjusting *p*-values for multiple comparisons. This approach was adopted as the LDA score cutoff serves to reduce false positives by setting an effect size threshold, balancing statistical significance with biological relevance [73].

#### Co-occurrence network analysis

To investigate the interaction of bacterial communities in non-diseased and diseased samples, a bacterial ecological co-occurrence network was constructed using the microeco R package v1.8.0 [75]. Low-abundance taxa were filtered out, retaining only ASVs with a relative abundance greater than 1% at any sample and present in 30% of samples. The co-occurrence relationships were calculated using the *WGCNA* R package v1.72.5 [76] based on a Spearman’s correlation coefficient (ρ) with a cut-off of 0.6 and a significance threshold of *p* < 0.001 for showing only stronger correlations. To control the false discovery rate (FDR) for multiple comparisons, the Benjamini-Hochberg (BH) adjustment method was used to adjust the *p*-value. An undirected co-occurrence network was then generated using the igraph R package and visualised using Gephi software (version 0.10) following the Circular network layout. Network topologies such as clustering coefficient, average degree, network diameter and average path length were calculated and compared between diseased and non-diseased gill and skin swabs.

To assess the keystone taxa in diseased and non-diseased gill and skin swab samples, degree and betweenness centrality were calculated for each node. A degree in network analysis implies the number of connections a taxon has in the network; a high degree is known as a “hub” and can be considered important due to higher connections. Betweenness centrality evaluates the extent to which a node acts as a bridge by measuring the shortest paths that pass through it. This metric highlights that a node plays a crucial role in connecting other nodes within the network, thereby identifying “keystone taxa” with significant influence on network organisation.

## Results

### Sequencing outputs

After thorough quality filtering and the removal of low-quality reads, a total of 37,427,997 high-quality sequence reads for the prokaryotic dataset across 298 samples and 1,356,961 high-quality reads for the micro-eukaryotic dataset across 57 samples were retained. This resulted in an average read depth of 125,597 sequences per sample for prokaryotes and 23,806 sequences per sample for microeukaryotes. In total, 32,127 ASVs were detected in the prokaryotes dataset (16S) with a total of 60 phyla and 2,237 genera. For the microeukaryotes (18S), 2,961 ASVs were identified, resulting in 26 divisions and 485 genera. Detailed sequencing profiles for each sample type are available in Supplementary Additional file 2, Table S2.

### Microbial compositions of tilapia gill, skin, and pond water

**Fig. 1:**
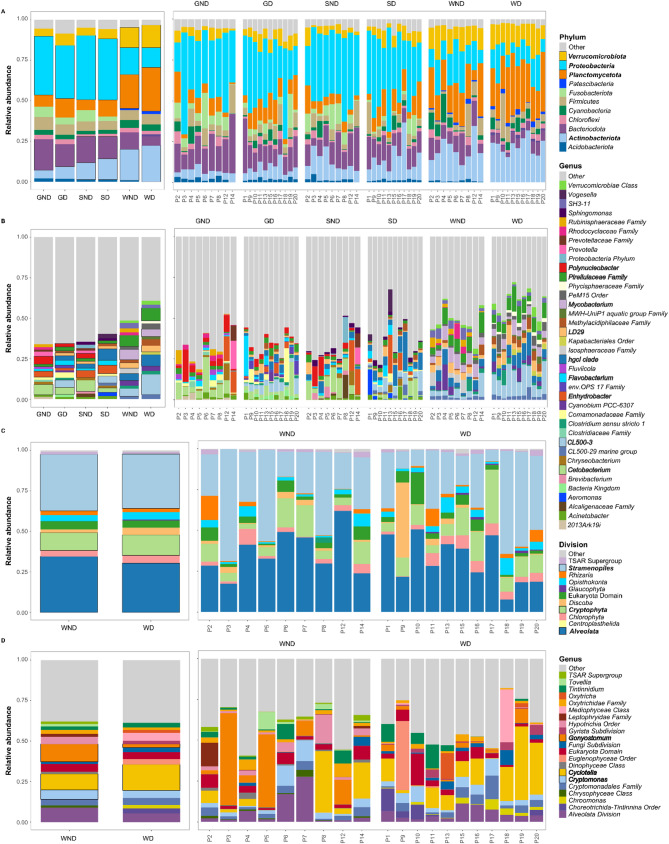
Microbial compositions in non-diseased and diseased ponds across various sample types. The stacked bar plots show the mean relative abundance of the top ten bacterial phyla (**A**), top 15 bacterial genera (**B**), top ten microeukaryotic divisions (**C**) and top 15 microeukaryotic genera (**D**). P1-P20 represent the 20 sampled ponds. The plots in the left panel display the mean abundance of each taxon across sample types (e.g.GD, GND, SD, SND, WD, WND). The right-side panel displays the relative abundances of taxa within each pond, with samples from the same pond grouped together to illustrate pond-specific community profiles. GD = Gill (Diseased); GND = Gill (Non-diseased); SD = Skin (Diseased); SND = Skin (Non-diseased); WD = Pond water (diseased); WND = Pond water (Non-diseased)

The microbial communities of tilapia gill, skin, and pond water were compared to assess which taxa were associated with the different sample types. The most dominant phyla in tilapia gill were *Proteobacteria* (34.69%) and *Bacteroidota* (16.95%), in the skin, *Proteobacteria* (38.80%), *Bacteroidota* (14.07%), and *Actinobacteriota* (12.30%), and in the pond water *Planctomycetota* (23.97%), *Actinobacteriota* (20.84%), *Proteobacteria* (14.63%%), and *Verrucomicrobiota* (13.38%) (Fig. 1A). At the genus level, *Cetobacterium* (5.62%), *Polynucleobacter* (3.82%), and *Flavobacterium* (2.95%) were abundant in tilapia gill; hgcI clade (5.11%), *Cetobacterium* (4.61%), *Enhydrobacter* (3.81%) and *Polynucleobacter* (2.94%) were abundant in tilapia skin; and CL500-3 (8.93%), unclassified *Pirellulaceae* (7.13%), hgcI clade (5.58%), *Mycobacterium* (5.40%) and LD29 (3.72%) were dominant in pond water (Fig. 1B). The microeukaryotic communities in pond water samples were dominated by Stramenopiles (36.92%), *Alveolata* (31.73%), and *Cryptophyta* (10.47%, Fig. 1C) divisions; and *Cyclotella* (13.82%), *Gonyostomum* (7.22%), and *Cryptomonas* (4.71%, Fig. 1D) genera.

**Fig. 2:**
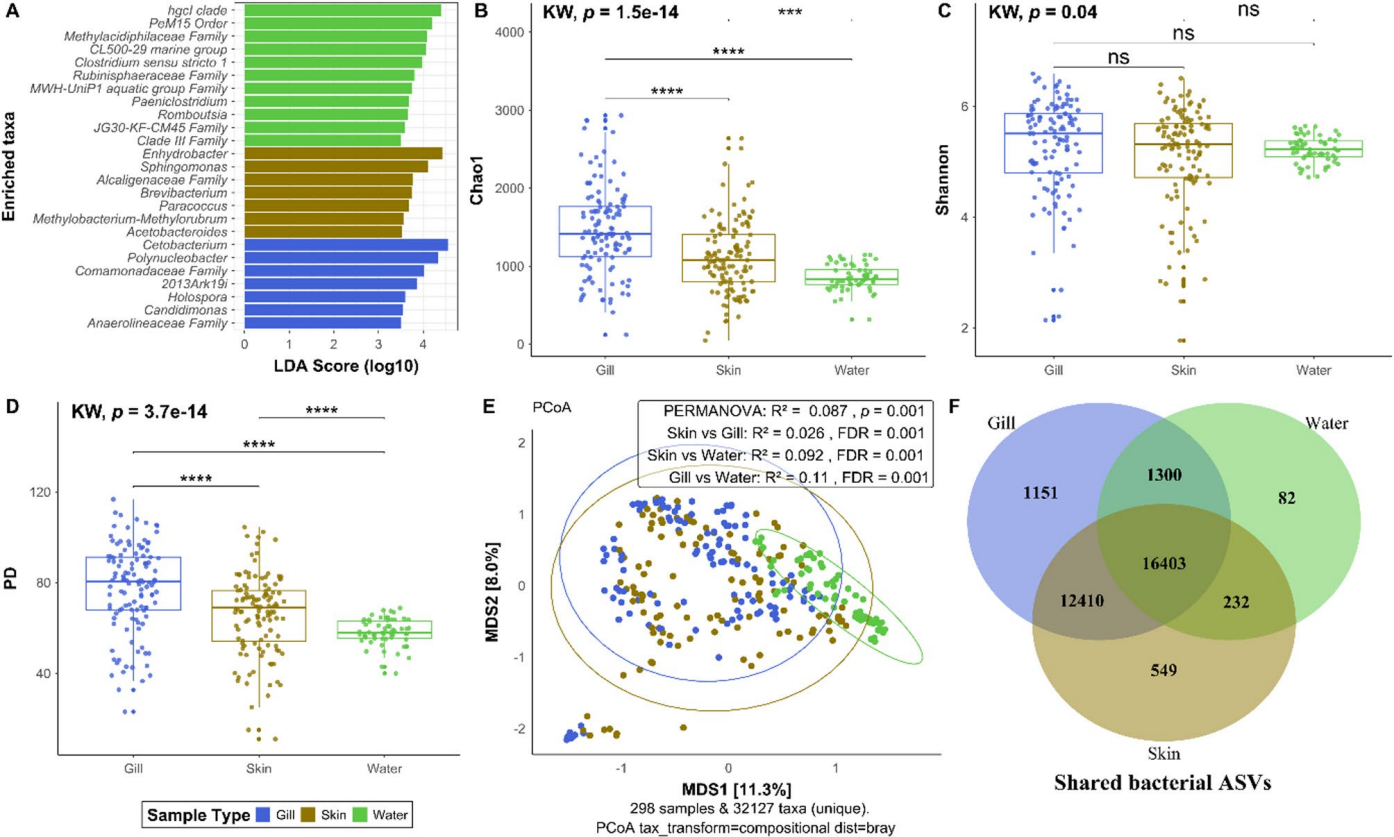
Differentially abundant taxa, bacterial diversity and shared ASVs between tilapia gill, skin and pond water. **A**) LEfSe characterisation of microbial communities across fish gill, skin and pond water. Bar chart showing the LDA score (log-transformed) of bacterial taxa that were found to be significantly different in tilapia gill or, skin or water compared with the other sample types. **B**) Chao1, **C**) Shannon, and **D**) Faith’s PD indices showing alpha diversity for the different sample types. **E**) PCoA ordination plot of beta diversity based on Bray-Curtis distance between different sample types. **F**) Venn diagram showing the number of shared and unique ASVs between tilapia gill, skin and pond water. KW = Kruskal-Wallis test. Asterisks indicate statistically significant differences in pairwise comparisons (ns: not significant; *: FRD < 0.05; **: FDR < 0.01; ***: FDR < 0.001; ****: FDR < 0.0001)

To identify taxa with a strong association with fish gill, skin and pond water, a linear discriminant analysis (LDA) effect size (LEfSe) analysis was performed. LEfSe analysis allows us to pinpoint microbial taxa that are significantly different between different biological groups by quantifying their effect size and highlighting the strength of association of each taxon with that specific environment. The effect size indicates which taxa are not only statistically significant but also biologically relevant as potential biomarkers for each habitat. The LEfSe results revealed that specific taxa were enriched across these sample types: genera *Cetobacterium*,* Polynucleobacter*,* Holospora*, and unclassified *Comamonadaceae* family were enriched in tilapia gill samples with the highest effect size (Fig. 2A). Skin samples showed enrichment of *Enhydrobacter*, *Sphingomonas*, unclassified *Alcaligenaceae* family, and *Methylobacterium-Methylorubrum*. Water samples were predominantly enriched with unclassified and/or candidate taxa, including the hgcl clade, PeM15 order, *Methylacidiphilaceae* family, and CL500-29 marine group (Fig. 2A).

### Microbial diversity of tilapia gill, skin, and pond water

To characterise within sample microbial diversity across tilapia gill, skin and pond water microbiomes, alpha diversity was calculated using Chao1, Shannon, and Faith’s PD indices. Based on Kruskal-Wallis (KW) tests, the bacterial alpha diversity differed (Kruskal-Wallis, *p* < 0.05) across fish gill, skin, and pond water, with tilapia gill showing the highest species richness and phylogenetic diversity (Fig. 2B-D). Application of a linear/generalised mixed effects model (alpha diversity ~ Sample type × Reported disease + Upazila + (1 | Pond)) revealed that bacterial alpha diversity measured by Chao1 and Faith’s PD significantly differed (*p* < 0.0001) across different sample types (Fig. 2B, D; Table 1). Pair-wise comparisons (through KW and LMMs) between the three sample types also showed significant variation in species richness (Chao1) and phylogenetic diversity (*p* < 0.0001, Fig. 2B, D, Additional file 2, Table S3). Species richness and phylogenetic diversity (PD) were highest in the tilapia gill, followed by the skin, and lowest in the pond water (Fig. 2B). For the Shannon diversity, although KW test illustrated a significant overall difference in alpha diversity across sample types (Kruskal-Wallis, *p* < 0.05, Fig. 2C), applying the Dunn post-hoc test found no significant differences between any specific pairs of sample types (gill vs. skin, gill vs. water, skin vs. water) (Fig. 2C; Table 1). The LMM that accounted for the repeated sampling within ponds, however, revealed no significant difference (Table 1), which may suggest that the observed difference in Shannon index across sample types may be due to pond level variation, which was not accounted for in the KW test.

To assess for possible relationships between alpha diversity metrics in the tilapia gill and skin, and pond water microbiomes within each pond, the mean Shannon diversity, Chao1 richness, and PD values for each pond were compared between sample types using a Pearson correlation test. Based on the Pearson correlation test, species richness (*R* = 0.69, *p* < 0.001), Shannon diversity (*R* = 0.71, *p* < 0.001), and phylogenetic diversity (*R* = 0.76, *p* < 0.0001) were strongly positively correlated between tilapia gill and skin but not between gill and water, or skin and water (Additional file 1, Fig. S3). Overall, based on the alpha diversity indices, significant differences were identified in bacterial richness, evenness and phylogenetic diversity between the three sample types.

**Table 1:** Summary of microbial alpha and beta diversity statistics assessing the effect of sample type, disease condition and geographical location

Index	Coefficient	Prokaryotes		Microeukaryotes	
		Chisq	*p*-value	Chisq	*p*-value
Chao1	Intercept	31.121	**< 0.0001**	11.380	**0.001**
	Sample type	80.342	**< 0.0001**		
	Reported disease	3.325	0.068	0.058	0.809
	Upazila	16.905	**0.010**	7.503	0.277
	Sample type: Reported disease	3.476	0.176		
Shannon	Intercept	1177.160	**< 0.0001**	77.299	**< 0.0001**
	Sample type	4.198	0.123		
	Reported disease	3.574	0.059	0.581	0.446
	Upazila	55.024	**< 0.0001**	4.787	0.571
	Sample type: Reported disease	1.800	0.406		
Faith’s PD	Intercept	116.567	**< 0.0001**	22.248	**< 0.0001**
	Sample type	76.904	**< 0.0001**		
	Reported disease	8.428	**0.004**	0.039	0.843
	Upazila	27.662	**< 0.0001**	6.128	0.409
	Sample type: Reported disease	7.032	**0.030**		
**Index**	**Coefficient**	**R** ^**2**^	***p*** **-value**	**R** ^**2**^	***p*** **-value**
Bray-Curtis	Sample type	0.085	**0.001**		
	Reported disease	0.034	**0.001**	0.0419	1
	Upazila	0.144	**0.001**	0.2553	1

This table summarises the effects of sample type (gill, skin and water), reported disease state (diseased and non-diseased), and their interactions, and geographical locations on Chao1, Shannon, and Faith’s Phylogenetic Diversity (PD). The formula used for fitting the model for alpha diversity was: lmer or glmm(alpha indices ~ Sample type × Reported disease + (1 | Pond), data = alpha diversity). The formula for PERMANOVA was: adonis2(bray distance ~ Sample type + Reported disease + Upazila, data = metadata, permutations = 999, strata = metadata$Pond name). Similar models were used for microeukaryotes except for different sample types since only water samples were assessed for microeukaryotes

Significant predictors (*p* < 0.05) are shown in bold

To investigate microbial structure and variability across the different sample types, beta diversity was calculated at the ASVs level using the Bray-Curtis distance metric. Dissimilarity analysis (PERMANOVA) with both only sample types and multiple factors showed a significant difference between tilapia gill, skin and pond water samples (PERMANOVA: R^2^ = 0.09, *p* = 0.001; Fig. 2E; Table 1). Pairwise comparisons showed a significant difference in the microbial community between tilapia gill, skin and pond water, albeit these differences between gill and skin samples were relatively weak (PERMANOVA: R^2^ = 0.03, FDR = 0.001, Fig. 2E, Additional file 2, Table S4). The biggest difference seen was between gill and water samples (PERMANOVA: R^2^ = 0.11, FDR = 0.001, Fig. 2E, Additional file 2, Table S4). PCoA plots also illustrated gill and skin bacterial communities clustered more closely together than with pond water (Fig. 2E). In terms of ASVs, 16,403 ASVs were shared among the three sample types, accounting for over 91% (16,403/18,017) of the total ASVs in water (Fig. 2F). Tilapia skin and gill shared 28,813 ASVs, representing over 92% of the ASVs in gill, and 97% of the ASVs in skin. Additionally, 92–98% of the water ASVs were shared between the skin and gill. Despite this substantial overlap, 1151, 549 and 82 ASVs were unique to gill, skin and water, respectively (Fig. 2F).

### Disease-associated microbial assemblages in tilapia gill, skin and pond water

**Fig. 3:**
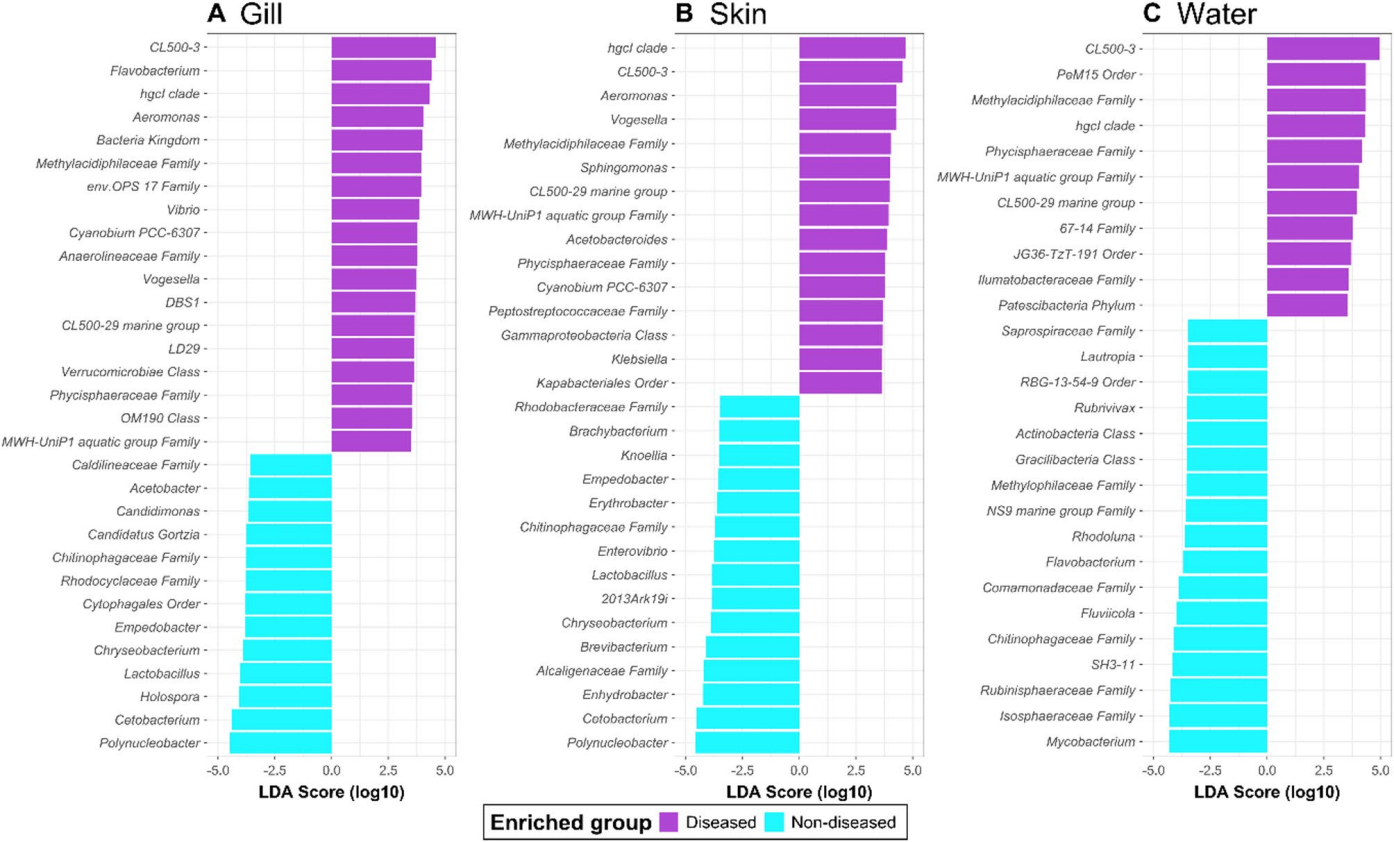
LEfSe characterisation of microbiomes between diseased and non-diseased sample types. Bar chart showing the LDA score (log-transformed) of bacterial taxa that were significantly different between diseased and non-diseased tilapia gill (**A**), skin (**B**) and pond water (**C**) by LEfSe (Kruskal-Wallis test, *p* < 0.05; Wilcoxon test, *p* < 0.05). Only Taxa with a LDA score greater than 3.5 are shown

Microbiota were compared between samples collected from the ponds with and without reported disease states to assess for differences due to disease. Figure 1 illustrates the taxonomic differences in the relative abundance of microbial communities (prokaryotes and microeukaryotes) between diseased and non-diseased sample types, highlighting specific taxa potentially associated with fish health and disease state. Differential abundance analysis using the LEfSe identified 31 taxa that were significantly different between diseased and non-diseased gill samples (Fig. 3A). Of these, in the gills of diseased fish, genera known to include pathogenic or opportunistic pathogens such as *Flavobacterium*, *Aeromonas*, *Vibrio*, and *Vogesella* were highly enriched (high LDA score). In contrast, genera as commensals for tilapia, such as *Cetobacterium*, were highly enriched in non-diseased gill samples (Fig. 3A). In non-diseased skin, beneficial/commensal bacterial genera such as *Cetobacterium*, *Lactobacillus*, *Brevibacterium* and *Polynucleobacter* were enriched, whilst in diseased skin samples, *Aeromonas*, *Vogesella* and *Klebsiella* were enriched (Fig. 3B). Except for *Flavobacterium*, none of the aforementioned genera was differentially abundant in the water between reported diseased and non-diseased ponds (Fig. 3C).

Microeukaryotic communities were investigated but only for the water samples. The dominant micro-eukaryotic divisions did not differ between diseased and non-diseased water samples and included *Stramenopiles* (35.01% − 39.38%), *Alveolata* (30.27% − 33.61%), *Cryptophyta* (09.07% − 11.56%) and *Discoba* (3.10% − 5.36%) (Fig. 1C). This was also the case at a lower taxonomic (genus) level, *Gonyostomum* (2.05% − 13.82%) and *Cyclotella* (9.65% − 17.08%) were the dominant genera in both diseased and non-diseased water samples (Fig. 1D).

**Fig. 4:**
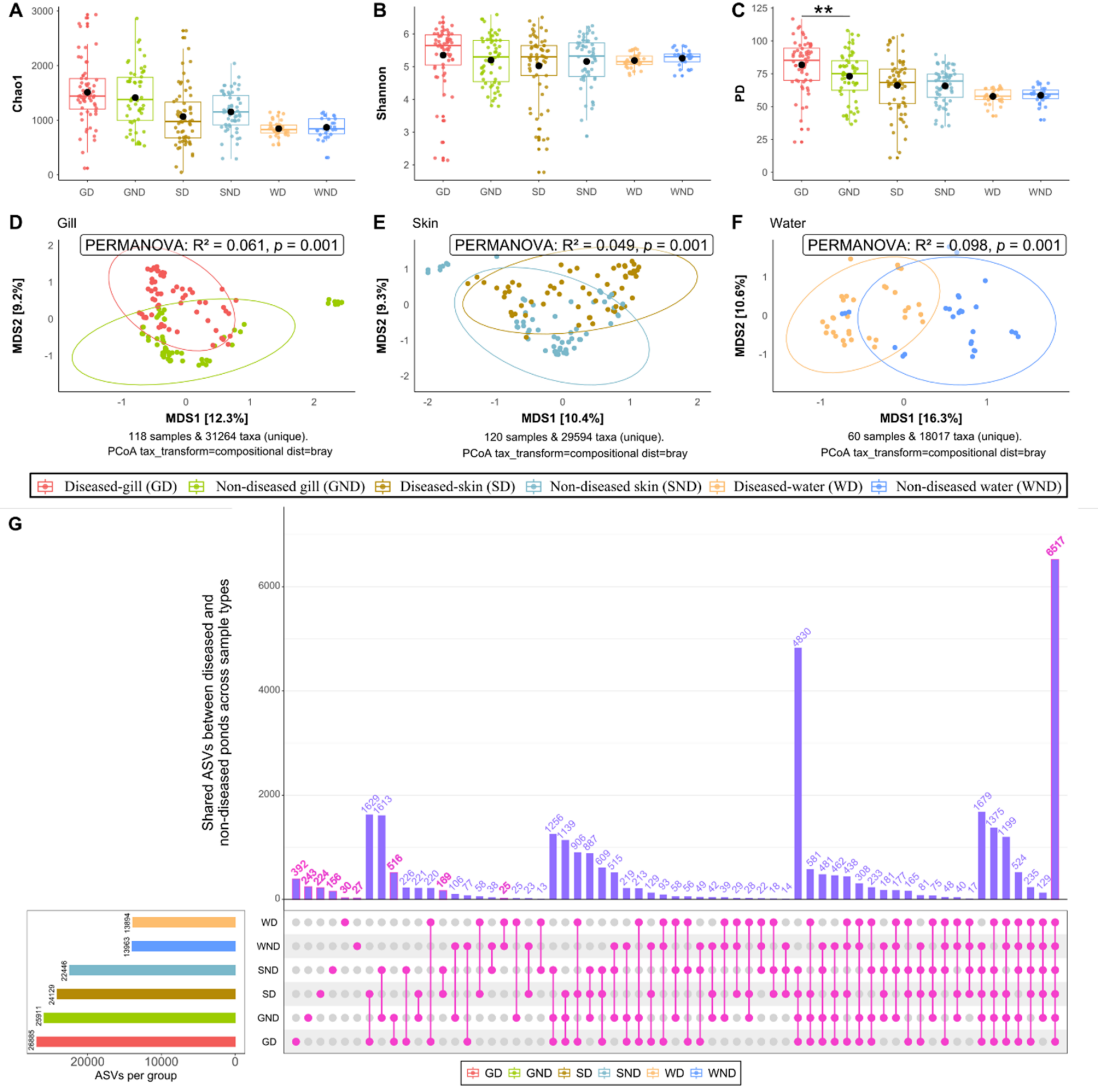
Bacterial diversity and shared ASVs between diseased and non-diseased tilapia gill, skin and pond water samples. **A**) Chao1, **B**) Shannon, and **C**) Faith’s PD indices for bacterial communities between diseased and non-diseased fish gills, skin, and pond water. The black dot represents the mean alpha diversity for each group (e.g. GD, GND, SD, SND, WD, WND). Beta diversity following Bray-Curtis distance on compositional ASVs and visualised with PCoA ordination showing bacterial structure between gill (**D**), skin (**E**) and water (**F**), respectively. **G**) Upset plot showing shared and unique ASVs between diseased and non-diseased fish gills, skin, and pond water. GD = Gill (Diseased); GND = Gill (Non-diseased); SD = Skin (Diseased); SND = Skin (Non-diseased); WD = Pond water (diseased); WND = Pond water (Non-diseased)

There were no significant differences in prokaryotic alpha diversity between the diseased and non-diseased samples within each sample category (i.e. between diseased and non-diseased gills, etc.), for Chao1 and Shannon indices (Kruskal-Wallis, *p* > 0.05; Fig. 4A-B). However, based on Faith’s PD index, there was a notable variation (Kruskal-Wallis, *p* < 0.01) between the diseased and non-diseased gill samples (Fig. 4C). The mixed-effects model showed a significant interaction between sample types and the reported diseased condition for PD (*p* < 0.05, Table 1), suggesting that disease condition influence the microbial phylogenetic diversity in different sample types. Post-hoc pairwise comparison between reported diseased and non-diseased samples within each sample type showed that reported disease was associated with an enrichment of phylogenetic diversity in diseased gill (Additional file 2, Table S3).

Beta diversity calculated using Bray-Curtis distance metric on compositional ASVs and visualised with PCoA ordination showed significant (albeit subtle) differences between diseased and non-diseased gill (PERMANOVA: R^2^ = 0.06, *p* = 0.001), skin (PERMANOVA: R^2^ = 0.05, *p* = 0.001), and pond water samples (PERMANOVA: R^2^ = 0.10, *p* = 0.001, Fig. 4D-F). The considerable number of taxa shared between diseased and non-diseased samples, however, shows they have similar bacterial community structures. Out of the 32,127 ASVs derived from all groups, 516 ASVs were shared between GD and GND, 169 ASVs were shared between SD and SND, 25 ASVs were shared between WD and WND, and 6,517 ASVs (20%) were common across all diseased and non-diseased gill, skin and pond water (Fig. 4G).

Based on both Kruskal-Wallis and the mixed effects models (alpha diversity indices ~ Reported disease + Upazila + (1 | Pond)), there were no significant differences (*p* > 0.05) in richness, diversity, or phylogenetic diversity of microeukaryotic communities between WD and WND (Additional file 1, Fig. S4A-C). Beta diversity analyses indicated significant differences (PERMANOVA: R^2^ = 0.06, *p* = 0.001) between water samples from diseased and non-diseased ponds when reported diseased was considered as a solo predictor (Additional file 1, Fig. S4D). However, after considering the geographical location and repeated sampling from the ponds, the effect of disease on microeukaryote diversity (alpha and beta) were not significant (*p* > 0.05, Table 1). Additionally, there is also a noticeable overlap between WD and WND samples with over 76% of ASVs shared between diseased and non-diseased water samples (Additional file 1, Fig. S4E).

### Differences in core taxa between diseased and non-diseased gill and skin microbiota

**Fig. 5:**
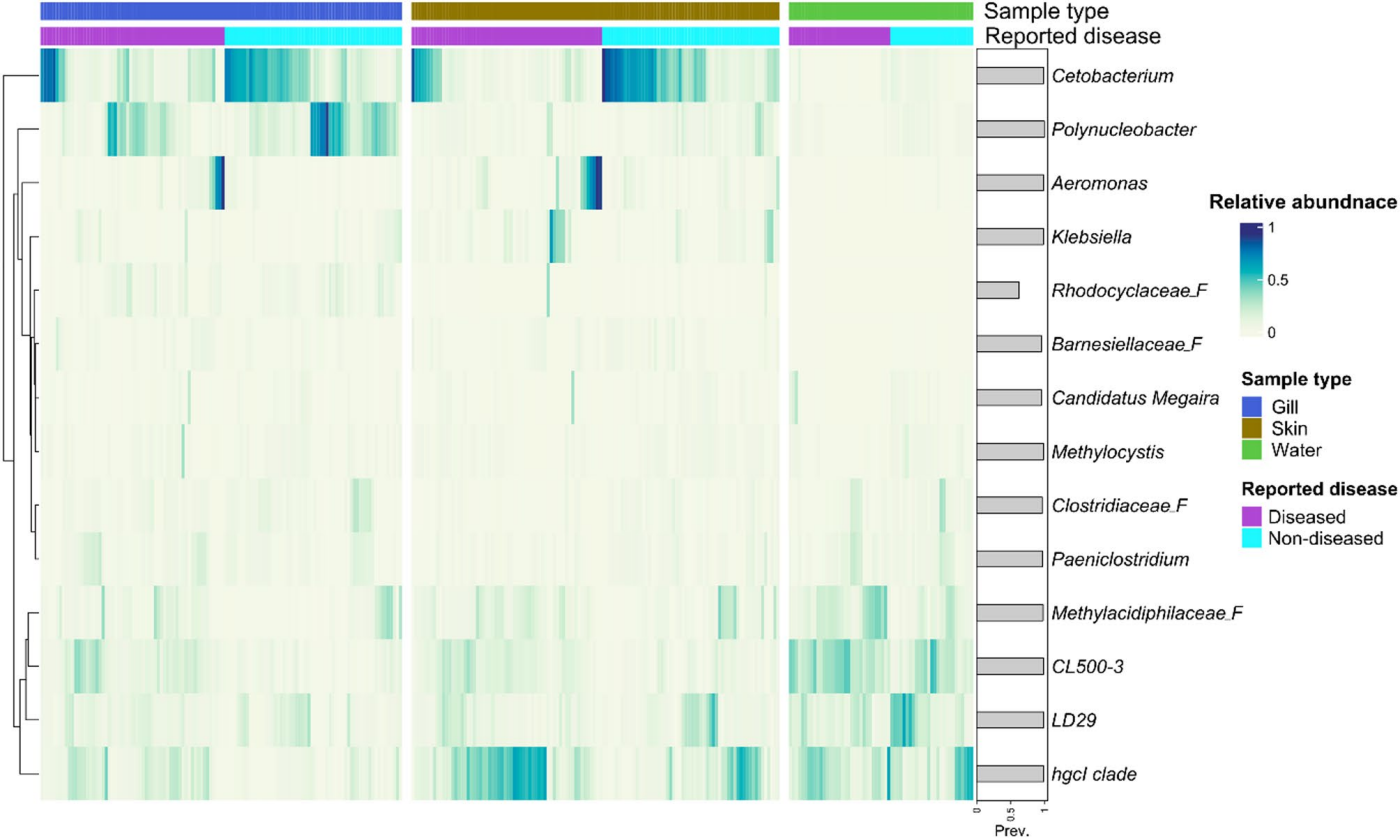
Core bacterial genera in diseased and non-diseased tilapia gill and skin, and pond water samples. Taxa with a relative abundance > 0.01% in at least one sample and a minimum prevalence of 90% (present in at least 90% of samples) were considered core microbiota, detected at the ASV level and aggregated at the genus level. Labels include genera and also the lowest available taxonomic rank e.g. “_F” represents taxonomic rank Family

To further identify microbial shifts associated with the health of the fish, the core microbiomes of diseased and non-diseased tilapia gills and skin were investigated. The core microbiota comprised 21 ASVs (belonging to seven phyla and 14 genera) in the gill, and 17 ASVs (belonging to seven phyla and 11 genera) in the skin (Additional file 2, Table S5). Of these core taxa, 15 ASVs were shared between the gill and skin; with six and two ASVs unique to the gill and skin, respectively (Additional file 2, Table S5). For the gill, of the core genera, *Cetobacterium* and *Polynucleobacter* had higher relative abundance in non-diseased gill compared to diseased fish, while in diseased gill *Aeromonas* had a higher relative abundance compared to non-diseased gills (Fig. 5). For the tilapia skin, of the core skin genera, *Cetobacterium* was higher in non-diseased skin, while *Aeromonas* and *Klebsiella* were dominant genera in diseased skin. All of these gill and skin core genera had a higher relative abundance compared to pond water. The unclassified CL500-3 and LD29 genera were abundant in pond water compared to the tilapia gill and skin core microbiota.

### Networks of microbial communities in diseased and non-diseased gill and skin

**Fig. 6:**
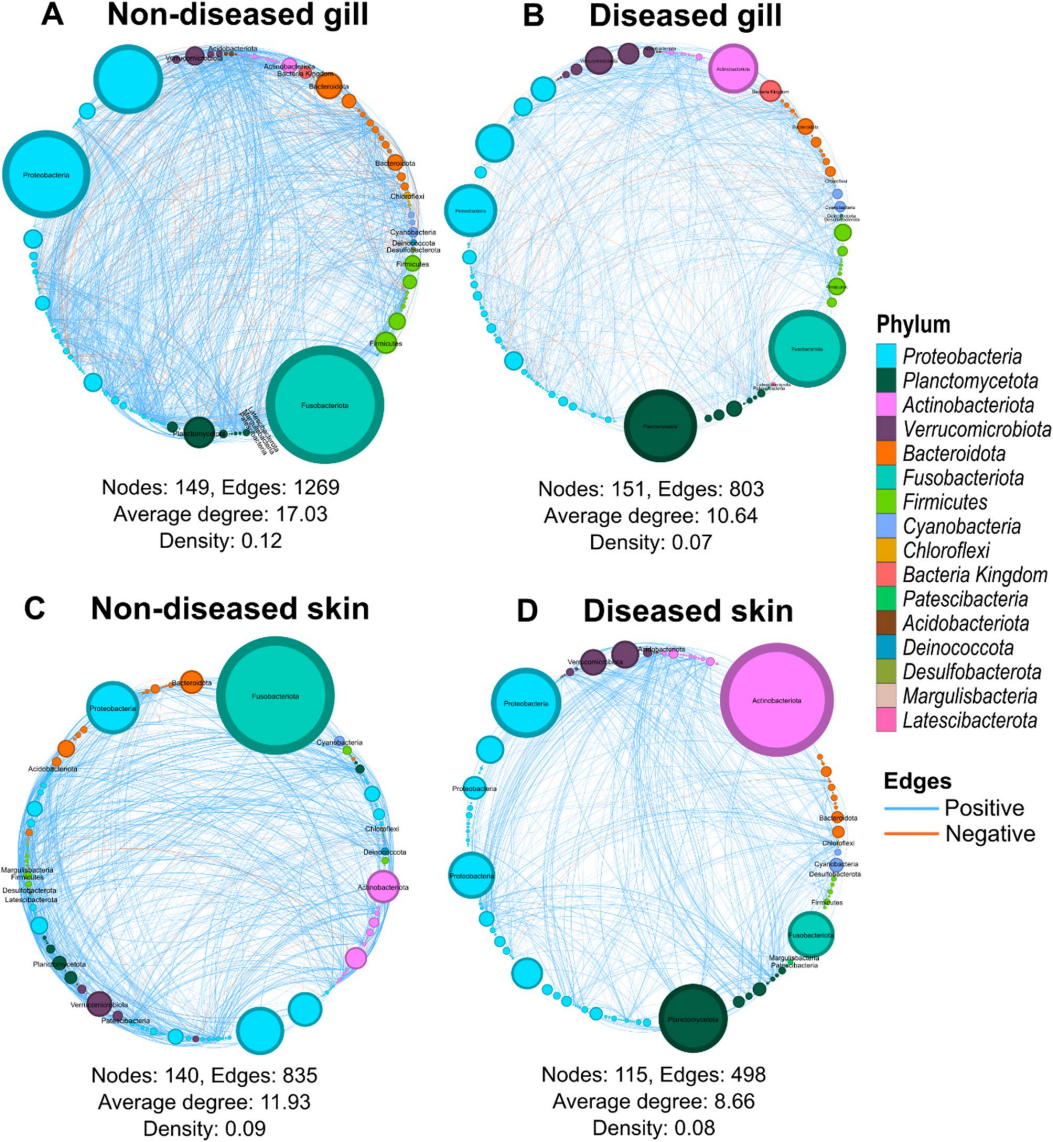
Co-occurrence network of bacterial communities in non-diseased and diseased gill and skin swab samples. Each node represents a genus, and edges (lines connecting nodes) represent significant correlations (Spearman’s correlation coefficient ρ > 0.6 and *p* < 0.001) between genera. The size of each node represents the relative abundance of the genus and genera belonging to a phylum are represented with a similar colour. Edges colour indicates positive (light blue) and negative (orange) correlations and weight (thickness) indicates the strength of the association between the taxa, the thicker the edges, the stronger the correlations

Network analysis revealed that the non-diseased gill microbial network had a higher number of edges (the connection between nodes, 1,269 vs. 803), average degree (number of connections for each node, 17.03 vs. 10.64), and density (indicating the level of interconnections within a network is, 0.12 vs. 0.07) compared with diseased gill (Fig. 6A-B, Additional file 2, Table S6). Similarly, non-diseased skin also had a greater number of nodes (taxon: 140 vs. 115), edges (835 vs. 498), average degree (11.93 vs. 8.66), and density (0.09 vs. 0.08) compared with the diseased skin microbial network (Fig. 6C-D, Additional file 2, Table S6). This indicates that non-diseased (for both gill and skin) microbial networks have a greater complexity and stronger microbial interactions compared with tissues from diseased fish. The proportion of positive edges was similar between diseased and non-diseased gill networks (89.41% vs. 89.68%) but was higher in diseased skin networks compared with non-diseased networks (99.2% vs. 94.97%; Additional file 2, Table S6).

In terms of microbial taxa, major phyla were similar in all networks except for diseased skin, where *Patescibacteria* and *Deinococcota* were absent (Fig. 6D). Betweenness centrality measures how often a node (taxon) is found on the shortest path connecting other nodes and the highest betweenness centrality is considered to act as “bridges” between different parts of the network. Thus, these are referred to as gatekeepers that maintain the communication, integrity and function of microbial communities [77]. The node with the highest degree may be considered as a hub and both node degree and betweenness centrality indicate the keystone species of that environment. The combination of either the highest Betweenness Centrality and Degree identified *Sphingobacterium* and *Flavobacterium* in non-diseased gill; *Cylindrospermopsis* and *Paeniclostridium* in diseased gill; *Flavobacterium* and *Candidatus Methylopumilus* in non-diseased skin; and *Cylindrospermopsis* and unclassified SH3-11 (*Verrucomicrobiota* phylum) as the keystone taxa (Additional file 1, Fig. S5, Additional file 2, Table S7). Of the top keystone taxa identified in each network (Additional file 2, Table S7), genera including *Sphingobacterium*, *Flavobacterium*, *Candidatus Methylopumilus*, unidentified *Isosphaeraceae* family and *Verrucomicrobiae* class were found to be common between the non-diseased gill and skin microbial network. The classes *Cylindrospermopsis*, SH3-11, and OM190 were common among the keystone taxa. The only keystone taxa common between the diseased and non-diseased gill microbial network were unclassified *Isosphaeraceae*. Similarly, only one taxon (*Lautropia* genus) was common between the diseased and non-diseased skin microbial networks (Additional file 2, Table S7).

### Microbial variation across geographical locations

**Fig. 7:**
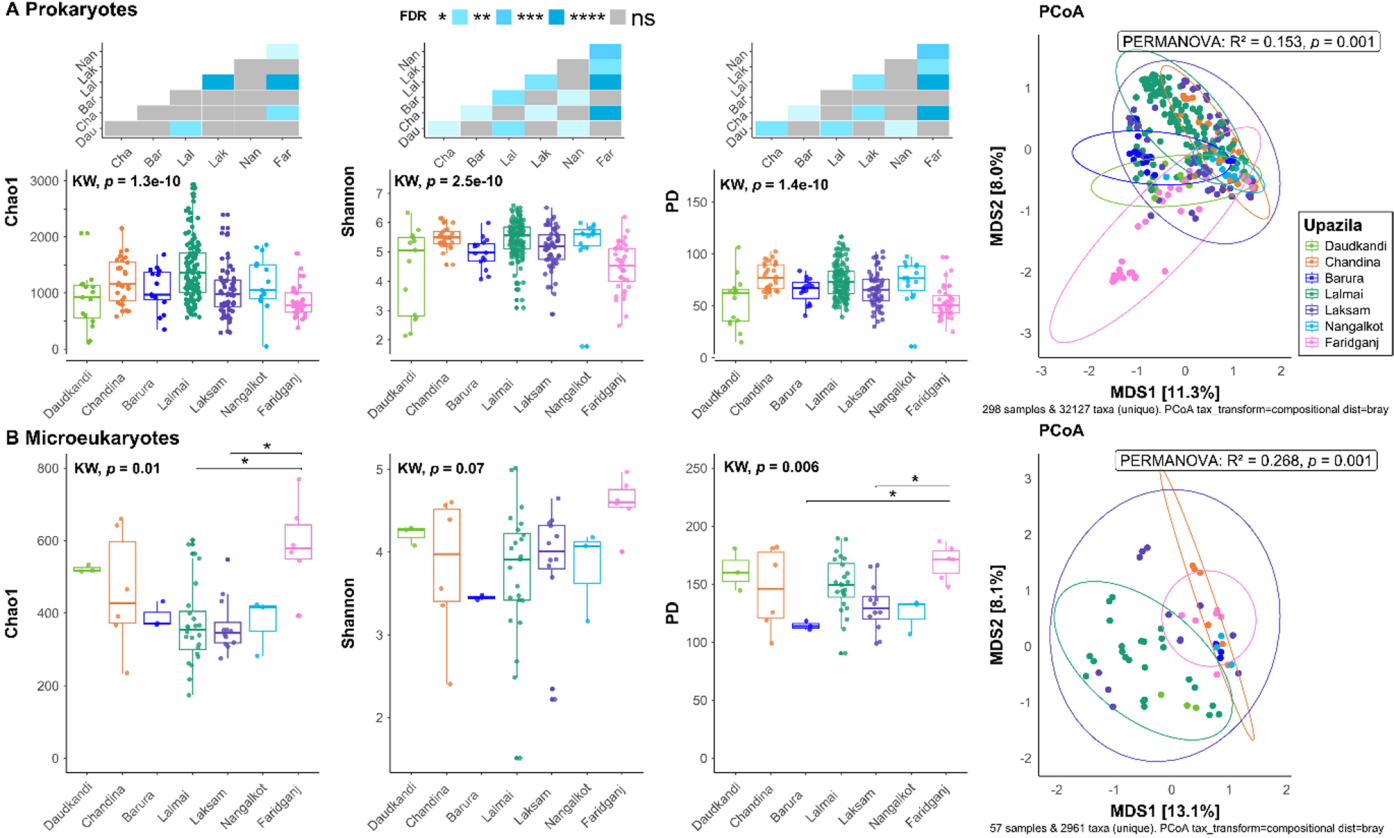
Alpha and beta diversity of prokaryotes (**A**) and microeukaryotes (**B**) across different geographical locations. Each dot represents a sample and is coloured by upazila. The heatmap shows pairwise comparisons of diversity indices between different upazila, highlighting the differences between them. On the x- and y-axis of the heatmap, the following abbreviations were used for upazilas: Dau = Daudkandi, Cha = Chandina, Bar = Barura, Lal = Lalmai, Lak = Laksam, Nan = Nangalkot, Far = Faridganj. In pairwise comparisons, asterisks (*: FDR < 0.05; **: FDR < 0.01; ***: FDR < 0.001; ****: FDR < 0.0001; ns: non-significant) represent statistically significant differences based on the false discovery rate (FDR)

Bacterial communities (alpha and beta diversity) varied significantly (Kruskal-Wallis, *p* < 0.0001 for all alpha diversity indices; PERMANOVA: R^2^ = 0.15, *p* = 0.001) among different upazilas (Fig. 7A). Pairwise comparison between different upazilas showed significant differences for all alpha diversity indices and beta diversity (Fig. 7A). A PCoA plot also indicated significant differences (PERMANOVA: R² = 0.15, *p* = 0.001) in prokaryotic community composition across different upazilas. In the mixed-effects models and PERMANOVA model that accounted for random variation across ponds and fixed effects of sample types, reported disease and geographical location, upazila remained as a significant predictor showing an overall significant variation of microbial alpha and beta diversity across upazilas (Table 1). However, pairwise comparison between upazilas showed a less consistent pattern for alpha diversity, especially for microbial richness and phylogenetic diversity (Additional file 2, Table S3). Pairwise comparisons for beta diversity, however, showed significant variation across different upazilas, with the highest variation observed between the Barura and Nangalkot upazila (PERMANOVA: R² = 0.28, FDR = 0.001) (Additional file 2, Table S4).

For the micro-eukaryotic communities, no significant differences were observed when applying the Shannon index. However, microbial richness (Chao1) and phylogenetic diversity were significantly different (Kruskal-Wallis, *p* < 0.05). In pairwise comparison, the richness of Faridganj upazila was found to be higher than Lamai and Laksam (Fig. 7B). The phylogenetic diversity (PD) of the Faridganj upazila was also found to be significantly higher than that of the Barura and Laksham upazilas (Fig. 7B). The PCoA plot indicated significant differences (PERMANOVA: R² = 0.27, *p* = 0.001) in microeukaryotic community composition (Fig. 7B). However, in the multifactor PERMANOVA model where reported disease and upazila along with repeated sampling from ponds were considered, no significant difference of geographical location (upazila) on the microeukaryotes community was observed on the beta diversity (Table 1).

## Discussion

The external mucosal surfaces (skin and gills) of fish are a crucial part of their defence system, acting as a natural barrier against invading pathogens and containing various immunogenic compounds [16]. Characterising the microbial composition and structure of external surfaces, therefore, is important for understanding both how microbial shifts relate to disease susceptibility and whether there are diagnostic features in these microbial alterations that can provide early warnings for disease onset. This study shows significant variations occur in the microbial compositions and diversity between external mucosal microbiomes of tilapia and pond water of semi-intensive polyculture in Bangladesh and evidence influence of reported disease on the external microbial structure of tilapia. Moreover, geographical location was found to have a significant influence on the microbial structure and diversity of tilapia.

Over 2000 classified, candidate and unclassified genera were identified in the 20 aquaculture ponds studied in Bangladesh, illustrating the highly diverse and complex nature of the microbial community in these aquaculture systems. These were mostly rare taxa with a few (around 21) abundant genera, the relative abundance of which ranged from 1 to ~ 9% (Fig. 1B). However, no single genus specifically dominated the microbial community in these aquaculture ponds. We show a significant difference between the fish’s external mucosal microbiomes and the pond water, as has been reported by others [18, 78–80]. It is not surprising given that the mucus-rich environments of these tissues provide a relatively higher amount of nutrition compared with the pond water environment. Unique ASVs in the different sample types and significant variation between the external mucosal microbiomes and pond water microbiomes indicate likely host-specific influence. The lack of a correlation between the species richness, phylogenetic diversity and overall diversity between the external mucosal (gill and skin) surfaces and pond water (Additional file 1, Fig. S3) suggests that although the pond water has a role in shaping the gill and skin microbiomes, the host has a strong influence on them also, again supporting other findings for tilapia and other fish species [21, 78]. Despite the differences in microbial diversity seen between the sample types, however, over 90% of the bacterial taxa in the pond water were shared with the tilapia gill and skin. This particularly high number of shared taxa between the ponds and external tissues may be explained by the fact that the ponds were largely static systems with very limited water exchange (depending on the water availability) and highlights the importance of pond water as the source of external mucosal microbiomes.

Findings from the current study also demonstrate overall significant variation in the microbial communities and structures between the different geographical locations (upazila), explaining 15% of the beta diversity for prokaryotic communities. Significant differences in microbial alpha diversity were also observed between different geographical locations (except for the Shannon index for microeukaryote communities). These differences across different geographical locations likely arise as a combination of differences in various biotic and abiotic factors, including stocking density, water physicochemistry, and the diet provided to the fish [18, 80, 81] as has been reported also for fish gut microbiomes [82, 83]. The observed bacterial phyla dominating the pond water (*Planctomycetota*, *Actinobacteria*, *Proteobacteria*, *Verrucomicrobiota*, and *Bacteroidota*) align generally with those reported in previous studies for tilapia cultures. However, *Firmicutes* was also found as a major phylum, as opposed to *Cyanobacteria* seen in other reported studies [21, 30, 84–86]. Again, this may be attributed to several factors, including different geographical locations presenting different environmental conditions. The presence of *Proteobacteria* and *Bacteroidota* as the major phyla occurring in tilapia gill and skin is consistent with previous reports for tilapia skin [38] and gill [21, 30].

Microbial dysbiosis, a change in microbial composition, diversity and predicted functions, of the skin and gill microbiome, has been reported previously in various fish species [25, 26, 29, 87, 88]. The pattern of dysbiosis of these microbial assemblages likely varies depending on the host species, tissue types and aetiological agent of diseases. In this study, although we saw no significant difference in alpha diversity indices (except for PD) between diseased and non-diseased gill, skin and water samples, there were significant, albeit weak, differences in the beta diversity between diseased and non-diseased samples for all sample types. A significant interaction between sample type and the PD may suggest that PD may respond differently across different sample types, but no difference after pairwise comparison between diseased and non-diseased samples within each sample type may indicate that the magnitude of this effect may be subtle.

There was significant variation in the relative abundance of certain genera between non-diseased and diseased samples, highlighting their potential role in fish health. Genera *Cetobacterium* and *Polynucleobacter* were differentially abundant in non-diseased gill and/or skin samples compared with their diseased counterparts. In the tilapia gut, *Cetobacterium* is a commensal known for its potential antagonistic activity against competing bacteria [89] and production of B12 that is essential for the metabolic function of the fish [90, 91]. It is also known as a major constituent of the skin [21, 30], gill and gut [91] of healthy tilapia [91]. Species of the genus *Polynucleobacter* are commonly found in freshwater ecosystems [92], and are known to play an important role in degrading organic matter and nutrient cycling [93]. There is no strong evidence for pathogenicity within these genera, and their role(s) are likely beneficial or neutral for fish health.

According to the aquaculture bacterial pathogen database, some members of the genera *Acinetobacter*, *Edwardsiella*, *Pseudomonas* (*Proteobacteria*); *Mycobacterium*, *Kocuria* (*Actinobacteria*); *Flavobacterium*, *Tenacibaculum*, *Chryseobacterium* (*Bacteroidota*); *Enterococcus*, *Lactococcus*, *Streptococcus* (*Firmicutes*) are potential fish bacterial pathogens [94]. Members of the genera *Streptococcus*, *Enterococcus*, *Aeromonas*, *Flavobacterium*, *Vibrio*, and *Edwardsiella* have been found to cause disease in tilapia (reviewed in [95]). All of these genera were observed in almost all sample types in the current study (Fig. 1B), although the relative abundance of most of them was less than 1%. Interestingly, the relative abundance of the diatom *Cyclotella* was almost double that in the ponds with diseased compared with non-diseased fish. Diatom *Cyclotella*, a key member of the phytoplankton community of aquatic ecosystems, and as an indicator of a changing environment [96] may in turn indicate a change in the overall health status of the pond ecosystem. It should be emphasised that the disease status of fish in the study ponds was poorly defined and studies where disease conditions are more overt (and well-characterised) will help better define signatures of the developing conditions of dysbiosis induced by disease and disease-forming states.

The external mucosal microbiome structure of tilapia will likely differ between disease and a healthy state. In an unhealthy/diseased state, pathogenic or opportunistic pathogens can become a major component of the microbial community, causing a diagnostic change in the microbial community structure. Identifying this, however, is complicated by the fact that the microbial community fluxes also occur in response to the changing physicochemistry of the pondwater conditions (and other factors). Nevertheless, some taxa make up what constitutes the core microbiome and play a vital role in the optimised microbiome functioning and stability [97]. Changes in the microbiome structure of these phyla are especially likely to hold the key to identifying changes associated with animals moving from a healthy to diseased state. In the tilapia gill and skin, 14 and 11 genera, respectively, were identified as constituting the core microbiome, some of which are consistent with previous reports for studies on tilapia [30, 98].

Our network analysis showed distinct structural differences between reported diseased and non-diseased gill and skin samples. The non-diseased samples had relatively more complex network structures compared to the diseased ones. This suggests that the bacterial communities in the non-diseased samples were more diverse and likely relatively more stable, which is consistent with previous findings reporting that stress (disease, antibiotic exposure) reduces the microbial network complexity and stability in the fish gut and pond water microbiome [99, 100]. A lower average degree and relatively less dense microbial network in the gill and skin samples from diseased fish also indicates a possible disruption in the bacterial community structure. Moreover, a higher positive edges proportion in the diseased skin samples network compared to the non-diseased skin also suggests a less stable microbial community structure.

Although networks featured similar phyla, the connections and distributions differed across them. In all networks, several taxa, including *Fusobacteriota*, *Actinobacteriota*, *Proteobacteria*, *Planctomycetota*, *Bacteroidota*, *Verrucomicrobiota*, and *Firmicutes* phyla appeared as large nodes. However, their relative abundance and connectivity varied between diseased and non-diseased networks for both gill and skin, suggesting their significance in both states. Additionally, some members of *Cylindrospermopsis* [101], *Enterobacterales* order (contains opportunistic pathogens such as *Escherichia coli* and *Aeromonas*), *Paeniclostridium* [102, 103], and *Paracoccus* [104] genera are known to be pathogenic to humans and/or animals. Thus, the presence of *Cylindrospermopsis* and *Mycobacterium* genera with high betweenness in diseased gill and skin networks suggests that these taxa could play a central role in the microbial structure, potentially through producing toxins or by pathogenic interaction within the microbial network. Furthermore, *Exiguobacterium*, which has been identified as a potential probiotic, was also present in diseased gills as a keystone species, and as such may serve to help stabilise the microbial community under stress.

Interestingly, in non-diseased gill and skin microbial networks, several pathogenic/opportunistic pathogenic genera (such as *Sphingobacterium* [105, 106], *Afipia* [107], *Aeromonas* [36, 108], *Flavobacterium* [109, 110], *Paraclostridium* [111, 112], and *Mycobacterium* [113]) were identified as keystone species. Even with a relative abundance of less than 1%, they may in turn play key ecological roles within the microbial community networks for maintaining network stability and resilience, consistent with other reports [114, 115]. The high betweenness centrality and high to moderate node degree indicate significant roles as connectors between different parts of the microbial network. These taxa may thus play an important role in modulating the enrichment of other taxa through competitive or antagonistic interactions, thus in turn affecting the stability of a microbial network. Change in environmental conditions e.g. water physicochemistry or host health, resulting in the proliferation of these genera may alter these interactions causing a shift toward pathogenicity. This monitoring of these keystone taxa (pathogenic) could help in early detection of microbial dysbiosis (imbalance) and network instability, which might cause disease outbreaks in the aquaculture system.

## Conclusions

This study observed a subtle, but nevertheless significant, difference in the microbiomes of gill and skin samples of tilapia from fish reported as diseased compared with those that were healthy, with a notable increase of pathogenic bacteria in the diseased samples, suggesting a possible dysbiosis associated with disease [34]. The bacterial genus *Aeromonas* (widely known to include opportunistic fish pathogens) was one of the most abundant core taxa in the gill and skin of tilapia in this study. However, pathogenicity is strain-specific, and our work was based on short hypervariable regions (V4), therefore, we cannot assert the pathogenic potential of these genera. It is noteworthy that opportunistic/pathogenic microbial communities were absent in the pond water samples, which may suggest that although the pond water may act as a conduit for the disease spread to the fish, it may not necessarily have a direct role in the disease manifestation in the animal itself. However, the microbial assemblage in the water could play a role in shaping the mucosal microbiome of diseased fish, in turn affecting disease susceptibility. The findings of this study underscore that tilapia skin and gill microbial community structures are sensitive to/indicative of host health status. The variation in microbial diversity across different upazilas, however, whereby local environmental conditions may play an important role in shaping the microbial communities in aquaculture ponds [18, 80, 81, 116] is an important (and complicating) consideration when assessing for microbiome features for disease diagnosis. Studies on how the microbiome shifts affect the functional role of the microbiome are much needed, as these will likely provide a more certain indicator(s) of fish health across diverse regions and environmental conditions and in turn, help diagnose and predict possible disease outbreaks in aquaculture systems.

## Supplementary Information

Below is the link to the electronic supplementary material.

Supplementary Material 1: Additional file 1: Supplementary figures. Fig. S1 Locations of the study area across different upazilas in Bangladesh. Fig. S2 Relative abundance of microbial taxa in ZymoBIOMICS Standard. Fig. S3 Pearson correlation between alpha diversity indices and tilapia gill, skin and pond water. Fig. S4 Microeukaryotic diversity and shared ASVs between water from the diseased pond and the non-diseased pond. Fig. S5 Keystone taxa analysis in microbial network.

Supplementary Material 2: Additional file 2: Supplementary tables. Table S1 Details of tilapia gill, skin and pond water sampled from earthen polyculture ponds in Bangladesh. Table S2 Sequencing profile for the prokaryotic (16S) and microeukaryotic (18S) datasets. Table S3 Pairwise comparison of alpha diversity assessing the effect of sample type, disease condition and geographical location. Table S4 Adonis pairwise comparisons of microbial structures calculated on Bray-Curtis dissimilarity across sample types, upazila, diseased and non-diseased samples within each sample type. Table S5 Bacterial taxonomic table of tilapia gill and skin-associated core microbiota. Table S6 Key attributes of co-occurrence networks. Table S7 The potential keystone genera in the diseased and non-diseased tilapia gill and skin microbial network.

## Data Availability

The 16S rRNA and 18S rRNA dataset(s) supporting the conclusions of this article are available in the European Nucleotide Archive (ENA) under BioProject accession number PRJEB85247. Data processing and analysis scripts are available at https://github.com/DebnathSanjit/Tilapia_skin_gill_microbiome_BD.

## Abbreviations

16S rRNA: 16S ribosomal ribonucleic acid
18S rRNA: 18S ribosomal ribonucleic acid
AIC: Akaike information criterion
ASV: Amplicon sequence variants
BIC: Bayesian information criterion
BH: Benjamini-Hochberg
DNA: Deoxyribonucleic acid
FDR: False discovery rate
GD: Gill-diseased
GND: Gill non-diseased
KW: Kruskal-Wallis test
LEfSe: Linear discriminant analysis (LDA) effect size
PCoA: Principal coordinate analysis
PCR: Polymerase chain reaction
PD: Phylogenetic diversity
PERMANOVA: Permutational multivariate statistical analysis of variance
SD: Skin-diseased
SND: Skin non-diseased
WD: Water diseased
WND: Water non-diseased
