# Supplementary material for: Skin and gill microbiome profiles and network structures in farmed tilapia (Oreochromis niloticus) and their relationships with health conditions
Source: Anim Microbiome. 2025 Oct 29;7:113. doi: 10.1186/s42523-025-00480-2 (PMC12574044; doi:10.1186/s42523-025-00480-2)
Supplement: Supplementary file 1 — Supplementary Material 1: Additional file 1: Supplementary figures. Fig. S1 Locations of the study area across different upazilas in Bangladesh. Fig. S2 Relative abundance of microbial taxa in ZymoBIOMICS Standard. Fig. S3 Pearson correlation between alpha diversity indices and tilapia gill, skin and pond water. Fig. S4 Microeukaryotic diversity and shared ASVs between water from the diseased pond and the non-diseased pond. Fig. S5 Keystone taxa analysis in microbial network. [file 42523_2025_480_MOESM1_ESM.docx]

**Skin and gill microbiome profiles and network structures in farmed tilapia (*Oreochromis niloticus*) and their relationships with health conditions.**

Sanjit C. Debnath^1,2,^**^†^**, Ashley G. Bell^3^, Jamie McMurtrie^1,2^, Ben Temperton^1^, Charles R. Tyler^1,2,^ **^†^**

1 Faculty of Health and Life Sciences, University of Exeter, Exeter EX4 4QD, Devon, UK

2 Sustainable Aquaculture Futures, University of Exeter, Exeter EX4 4QD, Devon, UK

3 Section of Nutrition, Department of Metabolism, Digestion and Reproduction, Faculty of Medicine, Imperial College London, Hammersmith Campus, Du Cane Road, London, W12 0NN

**†Correspondence**

scd226@exeter.ac.uk +44-(0)-1392-7654131

c.r.tyler@exeter.ac.uk +44-(0)-1392-7654131

**Submitted to:** *Animal Microbiome*

### Supplementary figures


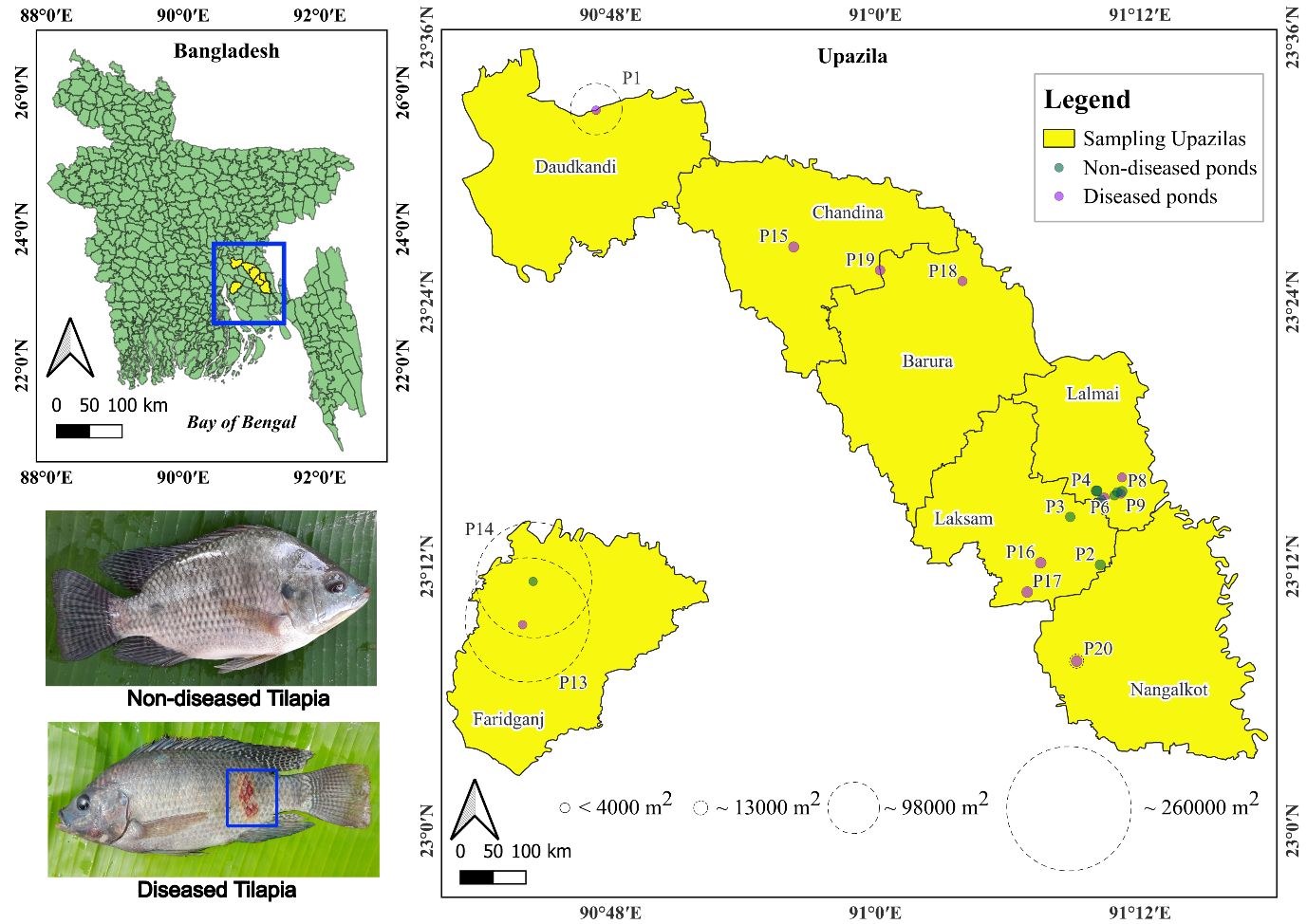


Fig. S1 Locations of the study area across different upazilas in Bangladesh.

Purple dots indicate reported diseased ponds and green dots indicate non-diseased ponds. Dots with solid lines represent pond size below 4000 m^2^ and dashed circles indicate the approximate size of ponds P1, P13, P14 and P20. The blue box on the tilapia fish demarks an example of lesions seen. On the skin of a diseased fish. The sampling location map was generated using the GPS coordinates using the QGIS free version (v 3.28.3).


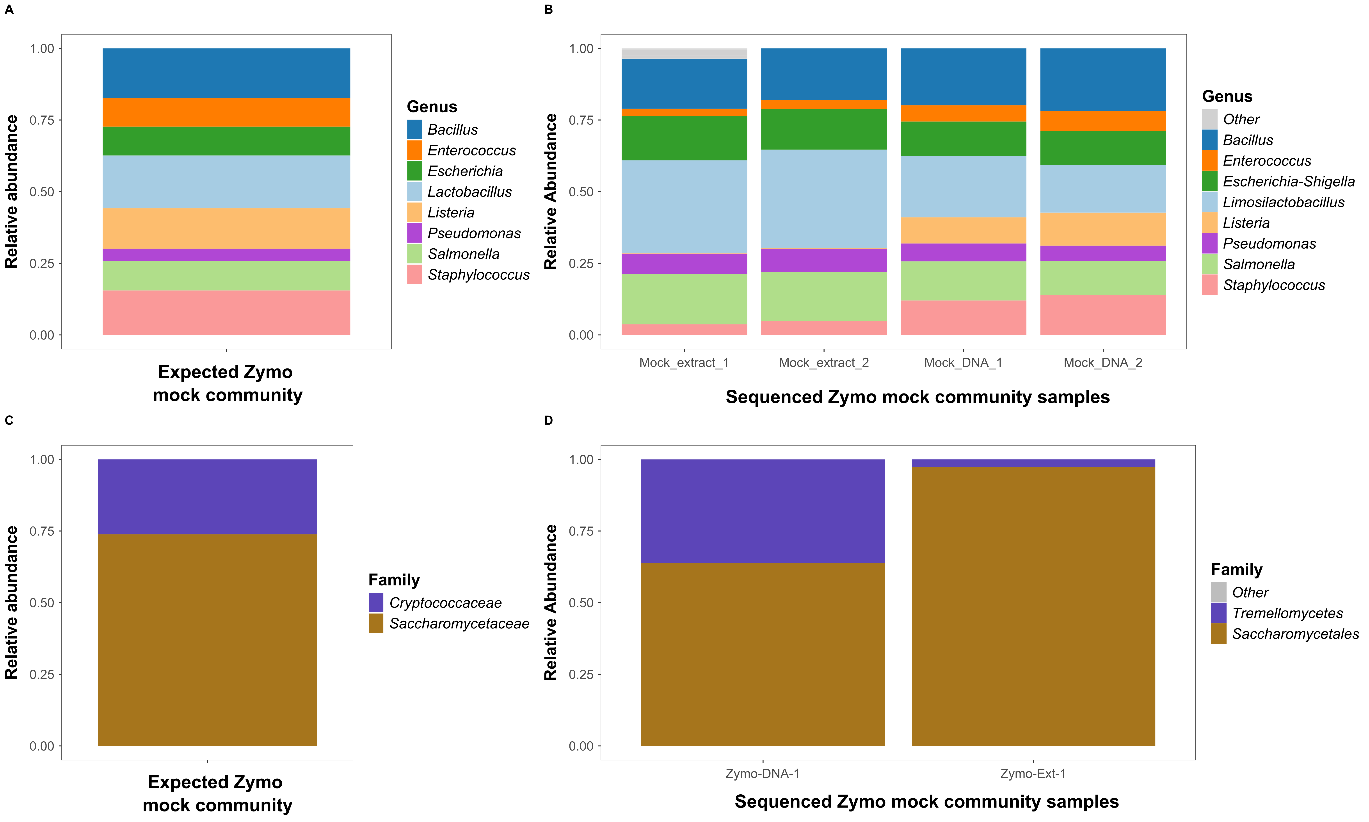


Fig. S2 Relative abundance of microbial taxa in ZymoBIOMICS Standard.

Expected relative abundance of 16S (A) and 18S (C) mock communities. Relative abundance of 16S (B) and 18S (D) communities in extracted (Mock_extract/Zymo-Ext) and standard ZymoBIOMICS mock DNA (Mock_DNA/ZymoDNA) used as a Positive control during PCR.


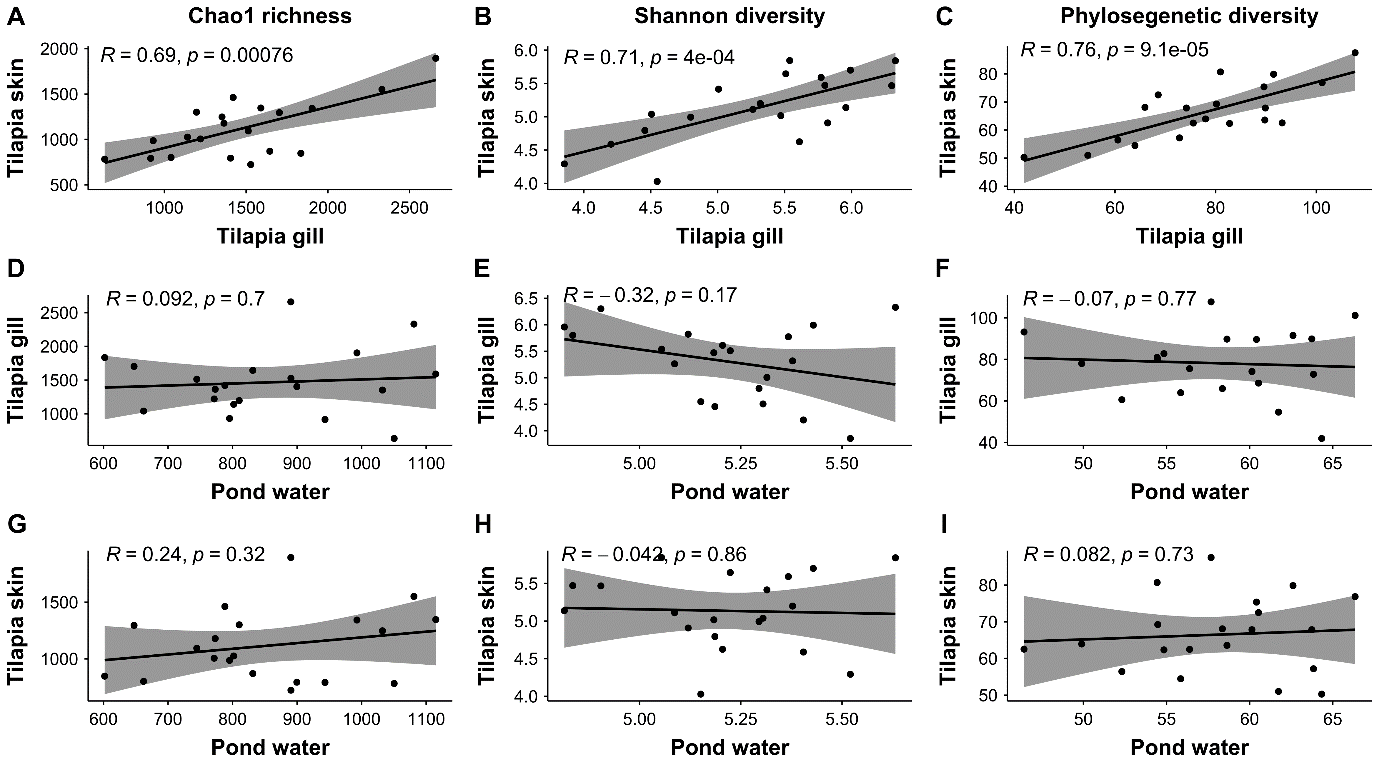


Fig. S3 Pearson correlation between alpha diversity indices and tilapia gill, skin and pond water.

The left panel shows a correlation of Chao1 richness between gill and skin (A), gill and water (D), and skin and water (G). The middle panel shows a correlation of Shannon diversity between gill and skin (B), gill and water (E), and skin and water (H). The right panel shows the correlation of phylogenetic diversity between gill and skin (C), gill and water (F), and skin and water (I). Each point represents the mean alpha diversity of each pond and the regression line indicates the Pearson’s correlation between alpha diversity and the various sample types. The grey-shaded area around the regression line represents Pearson’s correlation coefficient and is plotted with 95% confidence intervals.


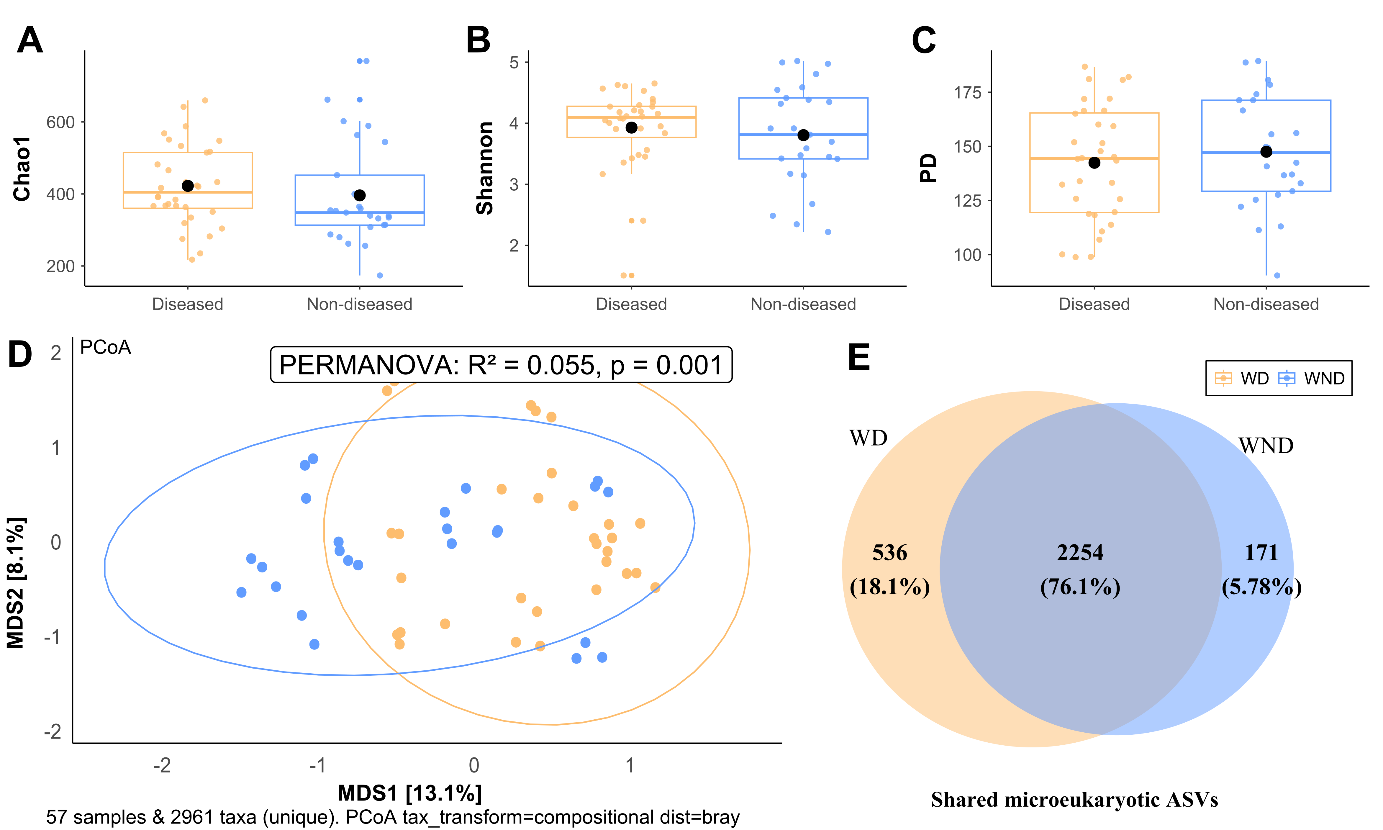


Fig. S4 Microeukaryotic diversity and shared ASVs between water from the diseased pond and the non-diseased pond.

A) Chao1 richness, B) Shannon diversity, C) Phylogenetic diversity between WD and WND. The black dot represents the mean alpha diversity for WD and WND. D) PCoA plot generated using Bray-Curtis at ASV level, E) Shared and unique microeukaryotic ASVs between WD and WND.


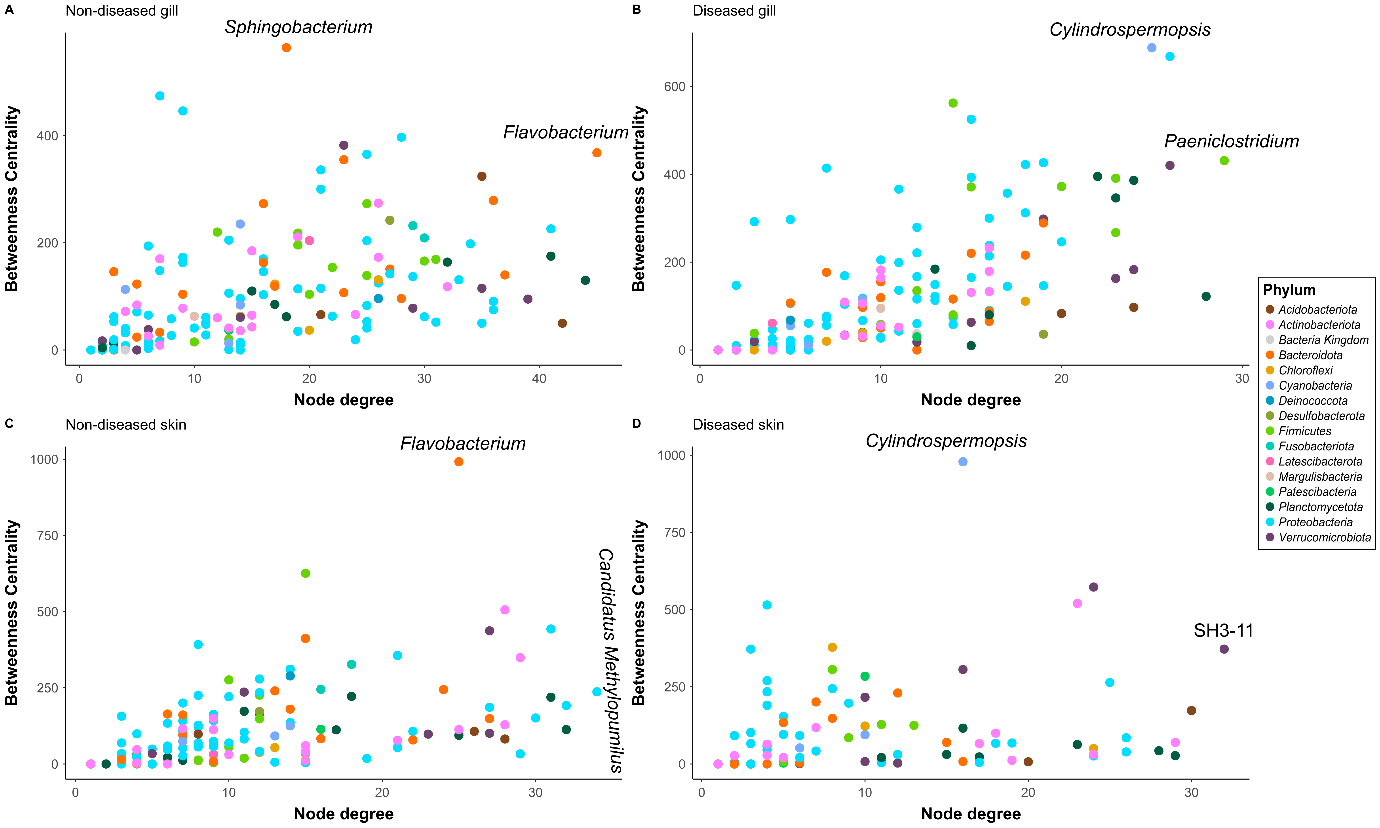


Fig. S5 Keystone taxa analysis in microbial network.

Betweenness centrality vs node degree of all taxa in non-diseased gill (A), diseased gill (B), non-diseased skin (C) and diseased skin (D) microbial network. A node with a high degree indicates a hub, and one with high betweenness centrality indicates a potential connector (bridge) between taxa in a network. Both measures were used as an indicator of potential keystone taxa and each dot (representing one genus) was coloured by the phylum they belong to. Genera with the highest in either property are highlighted in the plots.
